# Unrecognized diversity, genetic structuring, and phylogeography of the genus *Triplophysa* (Cypriniformes: Nemacheilidae) sheds light on two opposite colonization routes during Quaternary glaciation that occurred in the Qilian Mountains

**DOI:** 10.1002/ece3.10003

**Published:** 2023-04-19

**Authors:** Yan‐yan Du, Yan‐ping Zhang, Zhong‐yu Lou, Tai Wang

**Affiliations:** ^1^ Gansu Key Laboratory of Cold Water Fishes Germplasm Resources and Genetics Breeding Gansu Fisheries Research Institute Lanzhou China

**Keywords:** Continental River, cryptic species, DNA barcode, genetic differentiation, plateau loach

## Abstract

In recent years, the influence of historical geological and climatic events on the evolution of flora and fauna in the Tibetan Plateau has been a hot research topic. The Qilian Mountain region is one of the most important sources of biodiversity on the Qinghai‐Tibet Plateau. Many species existed in the region during the Pleistocene glacial oscillation, and the complex geographical environment provided suitable conditions for the survival of local species. The shrinkage, expansion, and transfer of the distribution range and population size of species have significant effects on genetic diversity and intraspecific differentiation. To reveal the effects of geological uplift and climate oscillation on the evolution of fish populations in the Qilian Mountains, we investigated the genetic structure, phylogenetic relationship, and phylogeographical characteristics of genus *Triplophysa* species in the Qilian Mountains using the mitochondrial DNA gene (COI), three nuclear genes (RAG1, sRH, and Myh6) and 11 pairs of nuclear microsatellite markers. We collected 11 species of genus *Triplophysa* living in the Qilian Mountains, among which *Triplophysa hsutschouensis* and *Triplophysa papillosolabiata* are widely distributed in the rivers on the northern slope of the Qilian Mountains. There was a high degree of lineage differentiation among species, and the genetic diversity of endemic species was low. The different geographical groups of *T. papillosolabiata* presented some allogeneic adaptation and differentiation, which was closely related to the changes in the river system. Except for the population expansion event of *T. hsutschouensis* during the last glacial period of the Qinghai‐Tibet Plateau (0.025 MYA), the population sizes of other plateau loach species remained stable without significant population expansion. Starting from the east and west sides of the Qilian Mountains, *T. hsutschouensis*, and *T. papillosolabiata* showed two species colonization routes in opposite directions. The geological events of the uplift of the Qinghai‐Tibet Plateau and the climatic oscillation of the Quaternary glaciation had a great influence on the genetic structure of the plateau loach in the Qilian Mountains, which promoted the genetic differentiation of the plateau loach and formed some unique new species. The results of this study have important guiding significance for fish habitat protection in the Qilian Mountains.

## Graphical abstract

There was a high degree of lineage differentiation among the *Triplophysa* species in the Qilian Mountains, and the genetic diversity of endemic species was low. The different geographical groups of *T. papillosolabiata* presented some allogeneic adaptation and differentiation, which was closely related to the changes in the river system. Except for the population expansion event of *T. hsutschouensis* during the last glacial period of the Qinghai‐Tibet Plateau (0.025 MYA), the population sizes of other plateau loach species remained stable without significant population expansion. Starting from the east and west sides of the Qilian Mountains, *T. hsutschouensis* and *T. papillosolabiata* showed two species colonization routes in opposite directions.

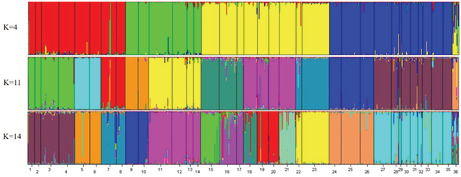

## 1 INTRODUCTION

Speciation is the result of a complex combination of factors that may accelerate or hinder speciation (Nosil et al., 2005). For example, different selection effects of different environmental conditions on populations can accelerate speciation (Wan et al., 2018; Wu et al., 2020; Zeng et al., 2020), and secondary contact of postglacial population dispersal can delay species differentiation (Hinojosa et al., 2019; Laurence, 2019). Geologic historical processes have a significant impact on species diversity distribution patterns (He et al., 2019; Zhang et al., 2019). Geomorphic changes caused by mountain uplift (Elias, 2009; Zhou et al., 2014) and climate change (Vila et al., 2005) will lead to population isolation, hinder genetic material exchange and increase differentiation among populations, which is also an important mechanism of speciation. Biodiversity was strongly influenced by global climate and environmental changes during the Quaternary glaciation, with repeated climatic oscillations leading to the extinction of many phylogenetic lineages, followed by recolonization during interglacial periods (Hewitt, 2000; Taberlet et al., 1998). Some cold‐tolerant species have been able to persist in glacial epoch and survive in refuges (Schmitt, 2007; Stewart et al., 2010), resulting in a high degree of species diversity in major refuge areas, whereas populations established during recolonization usually have low species diversity (Jablonski et al., 2016).

The unique geological features of the Tibetan Plateau make many areas a natural refuge for many species (Chen et al., 2017, 2018; Wang et al., 2018; Zhou et al., 2014). Qilian Mountain, located at the northeastern edge of the Qinghai‐Tibet Plateau, is a large and extremely important mountain range (Liu et al., 2002) that was uplifted along with the uplift of the Qinghai‐Tibet Plateau (Li, 1963; Li & Fang, 1998; Li et al., 2001). Three inland rivers were formed in the northern area of the Qilian Mountains: the Shiyang, Hei, and Shule Rivers. In the southern region of the Qilian Mountains are the Datong and Huangshui Rivers, which are important tributaries of the Yellow River basin. There is some debate about the uplift time of the Qilian Mountains, with some scholars suggesting that the uplift time was between 10 and 20 million years ago (MYA) (George et al., 2001; Wang, 1997; Yue et al., 2001), while some scholars believe that the uplift time began approximately 8.3 MYA (Fang et al., 2005; Li, 1963). The geological uplift process of the Qilian Mountains experienced many drastic climatic changes and at least two large glacial periods (Liu, 1946, 1951). These events had a great impact on the distribution pattern of species diversity from the Qilian Mountains (Zhao et al., 2011; Zhou et al., 2013). The biogeography of *Schizopygopsis chilianensis* (Li & Chang, 1974) (Cypriniformes: Cyprinidae), an endemic fish from the Qilian Mountains, suggests that this is a pattern of population diffusion along the Shiyang River system in the east of the Qilian Mountains to the west. In this process, species allopatronal evolution was related to the two great ice ages in the Qilian Mountains (Zhao et al., 2011).

*Triplophysa* Rendahl 1933 (Plateau loach) is a fish genus belonging to the family Nemacheilidae, included within the order Cypriniformes. This genus is widely distributed in the Tibetan Plateau and surrounding water systems and is a special group of loaches adapted to the cold environment of the Tibetan Plateau (Chen et al., 1996). Nine species of Plateau loach were first recorded in the Qilian Mountains (Zhu, 1989). With the discovery of new species and the redefinition of species, 11 species of plateau loach are reported in the Qilian Mountains (Wang, 1991; Wang, Yang, et al., 2015; Wang, Zhang, et al., 2015; Wu & Wu, 1992). Due to the narrow distribution range and difficulties in obtaining samples, some *Triplophysa* species were rarely investigated for a long time in the Qilian Mountains (Feng et al., 2017; He et al., 2011; Wang et al., 2020). Are there any hidden species taxa neglected in the Qilian Mountains? Meanwhile, what about the distribution ranges and population genetic structure of these plateau loaches in this mountain system. These issues are still unknown (Zhang et al., 2017).

In this study, we collected and analyzed data from the plateau loach fish (genus *Triplophysa*) *populations* in the Qilian Mountains, in order to: (1) estimate species diversity and species geographic distribution of the plateau loach fish; (2) reveal the implicit diversity of widely distributed species in the region; and (3) reconstruct the biogeographic history of the plateau loach fish population in the Qilian Mountains. Finally, we tested whether the species differentiation of the plateau loach in the Qilian Mountains was driven by specific geological events and whether the species distribution was likely to coincide with the changes in water systems in the Qilian Mountains. Therefore, we expected to suppose that the genetic diversity of the Plateau loach was related to the geological changes in the region.

## 2 METHODS

### 2.1 Sample collection

**FIGURE 1:**
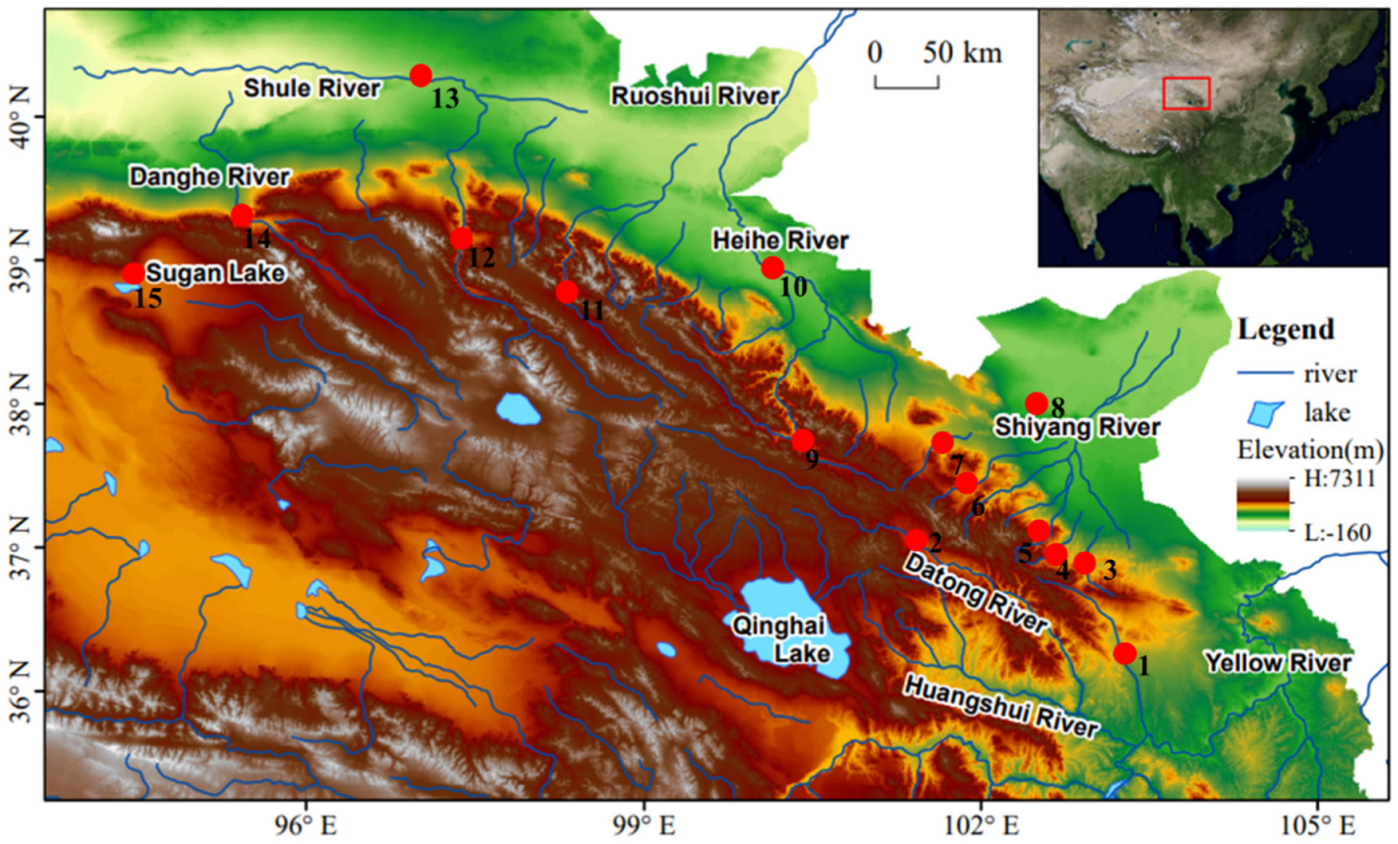
Sample collection of the *Triplophysa* fishes. Details of the 15 sites and collected specimens are provided in Table 1. (This base map is from 91 Vita Assistant software http://www.91weitu.com/index.htm, edited in Adobe Photoshop CS5 software).

In this study, a total of 548 plateau loach fish samples were collected from the Datong, Huangshui, Shiyang, Hei, and Shule Rivers in the Qilian Mountains from the years of 2017 to 2020 (Figure 1 and Table 1). As outgroup, nine *Hedinichthys yarkandensis* (Day 1877) were collected from the Shule River for further analysis. These specimens were captured using a cage net. Morphological identification of fishes is mainly based on taxonomic books (Kottelat, 2012; Wang, 1991; Zhu, 1989). The right pectoral fin strip of the specimen was cut and stored in 95% ethanol for total DNA extraction. The voucher specimen was fixed in 10% formaldehyde solution and stored in the fish exhibition room of Gansu Fisheries Research Institute.

**TABLE 1:** Genetic diversity information of the *Triplophysa* fishes.

Species	Location			Latitude and longitude	N	COI			Microsatellite				
	River system	Number	Location name			Nh	Hd	Pi	Na	Ne	Ho	He	PIC
*Triplophysa shiyangensis* (Zhao & Wang, 1983)	Shiyang River	4	Huangyanghe River	102°38.567′, 37°30.283′, 2180 m	23	11	0.81	0.00399	4.182	2.251	0.522	0.461	0.880
	Shiyang River	5	Zamuhe River	37°30.962′, 102°25.906′, 2626 m	9	6	0.889	0.00636	3.455	2.310	0.535	0.444	0.803
	Shiyang River	6	Huangcheng reservoir	38° 2.807′, 101°57.428′, 2465 m	8	4	0.643	0.00391	2.364	1.957	0.568	0.421	0.656
	Shiyang River	7	Xidahe River	38° 3.592′, 101°23.607′, 2835 m	21	10	0.758	0.00345	3.000	2.166	0.541	0.431	0.761
*Triplophysa stoliczkae* (Steindachner 1866)	Yellow River	1	Tianzhu	36°18.697′, 103°25.040′, 2078 m	15	6	0.762	0.00316	2.091	1.574	0.212	0.280	0.448
	Shiyang River	3	Shibalibao reservoir	37°22.566′, 102°55.645′, 2273 m	20	6	0.747	0.00225	2.364	1.789	0.323	0.336	0.554
*Triplophysa strauchii* (Kessler 1874)	Heihe River	9	Babaohe River	38° 1.717′, 100°34.948′, 3076 m	12	2	0.53	0.00088	3.636	2.452	0.576	0.528	0.934
	Heihe River	10	Heihelinze	39°08.376′, 100°23.839′, 1412 m	20	2	0.505	0.00084	3.727	2.551	0.586	0.529	0.962
*Triplophysa scleroptera* (Herzenstein 1888)	Yellow River	1	Tianzhu	36°18.697′, 103°25.040′, 2078 m	14	3	0.714	0.00155	1.500	1.181	0.043	0.127	0.205
	Yellow River	2	Datonghe River	37°27.445′, 101°25.495′, 2934 m	17	3	0.676	0.00161	1.000	1.000	——	——	——
*Triplophysa tenuis* (Day 1877)	Heihe River	10	Heihelinze	39°08.376′, 100°23.839′, 1412 m	19	5	0.532	0.00147	3.364	1.902	0.448	0.373	0.686
	Shule River	12	Changma	39°52.098′, 96°44.983′, 2054 m	31	3	0.4645	0.00083	3.636	2.105	0.478	0.430	0.787
	Shule River	13	Shuangta reservoir	40°33.417′, 96°19.2′, 1304 m	9	5	0.722	0.00184	2.818	1.930	0.455	0.376	0.649
	Shule River	14	Danghe River	39°32.795′, 94°50.900′, 2056 m	10	2	0.2	0.00033	2.364	1.773	0.473	0.369	0.596
*Triplophysa leptosoma* (Herzenstein 1888)	Shule River	12	Changma	39°52.098′, 96°44.983′, 2054 m	12	3	0.667	0.00126	2.546	1.844	0.398	0.407	0.656
*Triplophysa papillosolabiata* (Kessler 1879)	Shiyang River	7	Xidahe River	38° 3.592′, 101°23.607′, 2835 m	8	3	0.464	0.00083	2.546	2.050	0.568	0.489	0.756
	Heihe River	9	Babaohe River	38°01.717′, 100°34.948′, 3076 m	18	3	0.307	0.00088	3.909	2.181	0.535	0.488	0.882
	Heihe River	10	Heihelinze	39°08.376′, 100°23.839′, 1412 m	15	2	0.419	0.00069	3.727	2.429	0.533	0.530	0.941
	Shule River	11	Yanglong	38°48.872′, 98°24.702′, 3364 m	14	4	0.396	0.00071	3.273	2.150	0.539	0.501	0.850
	Shule River	12	Changma	39°52.098′, 96°44.983′, 2054 m	23	3	0.561	0.00185	4.727	2.571	0.629	0.578	1.056
	Shule River	14	Danghe River	39°32.795′, 94°50.900′, 2056 m	9	4	0.778	0.00249	3.182	2.472	0.647	0.587	0.948
	Shule River	15	Sugan Lakes	39°02.364′, 94°10.523′, 2807 m	22	4	0.714	0.00211	3.455	1.992	0.533	0.448	0.790
*Triplophysa wuweiensis* (Li & Chang 1974)	Shiyang River	7	Xidahe River	38° 3.592′, 101°23.607′, 2835 m	36	3	0.386	0.00086	2.727	1.945	0.462	0.352	0.618
*Triplophysa siluroides* (Herzenstein 1888)	Yellow River	2	Datonghe River	39°32.795′, 94°50.900′, 2056 m	16	6	0.735	0.00203	1.455	1.117	0.102	0.088	0.148
*Triplophysa robusta* (Kessler 1876)	Yellow River	1	Tianzhu	36°18.697′, 103°25.040′, 2078 m	18	11	0.915	0.00447	1.909	1.454	0.268	0.197	0.331
	Shiyang River	3	Shibalibao reservoir	37°22.566′, 102°55.645′, 2273 m	25	10	0.82	0.00365	2.000	1.471	0.280	0.208	0.357
*Triplophysa hsutschouensis* (Rendahl 1933)	Shiyang River	3	Shibalibao reservoir	37°22.566′, 102°55.645′, 2273 m	12	3	0.318	0.00111	2.636	1.977	0.356	0.371	0.628
	Shiyang River	5	Zamuhe River	37°30.962′, 102°25.906′, 2626 m	3	2	0.667	0.00221	1.818	1.596	0.303	0.321	0.427
	Shiyang River	6	Huangcheng reservoir	38° 2.807′, 101°57.428′, 2465 m	10	3	0.689	0.00151	2.000	1.658	0.346	0.295	0.460
	Shiyang River	7	Xidahe River	38° 3.592′, 101°23.607′, 2835 m	10	2	0.467	0.00077	3.091	1.841	0.346	0.343	0.626
	Shiyang River	8	Jinchuanxia reservoir	38°19.887′, 102° 1.632′, 1827 m	6	3	0.6	0.00144	2.909	1.839	0.485	0.430	0.704
	Heihe River	9	Babaohe River	38°1.717′, 100°34.948′, 3076 m	12	2	0.167	0.00028	1.636	1.127	0.068	0.086	0.159
	Heihe River	10	Heihelinze	39°08.376′, 100°23.839′, 1412 m	12	1	0	0	2.273	1.539	0.258	0.277	0.466
	Shule River	12	Changma	39°52.098′, 96°44.983′, 2054 m	24	1	——	——	2.273	1.377	0.221	0.226	0.398
	Shule River	13	Shuangta reservoir	40°33.417′, 96°19.2′, 1304 m	15	1	——	——	1.818	1.345	0.303	0.196	0.305
*Hedinichthys yarkandensis* (Day 1877)	Shule River	13	Shuangta reservoir	40°33.417′, 96°19.2′, 1304 m	9	1	——	——	2.818	2.059	0.404	0.460	0.745

Abbreviations: Hd, Haplotype diversity; He, mean expected heterozygosity; Ho, mean observed heterozygosity; N, Number of specimens; Na, Mean number of observed alleles; Ne, Mean number of effective alleles; Nh, Number of haplotypes; Pi, Nucleotide diversity; PIC, Polymorphic information index.

### 2.2 DNA extraction, amplification, and sequencing

Total genomic DNA was extracted from pectoral fins using a high‐salt method (Sambrook & Russell, 2001). A partial sequence of the mitochondrial cytochrome oxidase subunit 1 (COI) gene was amplified for all individuals (Ward et al., 2005). Three partial nuclear genes were amplified and sequenced: Recombinase‐activating gene 1 (RAG1), rhodopsin (sRH1), and myosin heavy polypeptide 6 (Myh6). The primer information and annealing temperature of PCR amplification conditions are shown in Table 2. The PCR amplification system for COI genes was constructed to be 30 μL, which contained 1.0 μL genomic DNA (100 ng/μL), 1.5 μL upstream and downstream primers (10 mM), 0.375 μL Taq DNA polymerase (Takara Biomedical Technology Beijing Co, Ltd), 1.5 μL dNTPs (2.5 mM), 3 μL 10× Taq buffer (Takara) and 21.125 μL double‐distilled water. The PCR amplification conditions included denaturation at 95°C for 3 min, denaturation at 95°C for 45 s, annealing at 53°C for 45 s, extension at 72°C for 45 s, 35 cycles, and denaturation at 72°C for 10 min. The PCR amplification system for three nuclear genes was constructed to be 25 μL, which contained 1.0 μL genomic DNA (100 ng/μL), 1.0 μL upstream and downstream primers (10 mM), 12.5 μL Taq enzyme premix (2 × Taq Master Mix, BioTeke Corporation, China) and 9.5 μL double‐distilled water. The PCR conditions were as follows: predenaturation at 94°C for 5 min, denaturation at 94°C for 30 s, annealing at 54–58°C for 30 s, extension at 72°C for 60 s, for a total of 30–35 cycles, and extension at 72°C for another 10 min after the reaction (Wang et al., 2020). PCR products were bidirectionally sequenced after passing 1% agarose gel electrophoresis detection using sequencing primers for amplification. The new sequences have been deposited in GenBank (accession numbers: OQ359293–OQ359372, OQ360026–OQ360052, OQ363686–OQ363749, OQ376574–OQ376597).

**TABLE 2:** Primers of mitochondrial DNA gene and nuclear gene sequences in this study.

Gene	Name of primer	Sequence of primer	Product size	*T*_m_ (°C)	References
COI	F1 R1	TCAACCAACCACAAAGACATTGGCAC TAGACTTCTGGGTGGCCAAAGAATCA	651 bp	53	Ward et al. (2005)
RAG1	RAG1‐F	CTGAATTCTTGTGAGCCTCCATRAAC	841 bp	54	Chen (2019)
	RAG1‐R	AGCTGCAGTCAGTAYCACAAGATGT			
sRH1	sRH1‐F	GTACGTCACCATCGAGCACA	699 bp	58	Feng et al. (2019)
	sRH1‐R	GCTGGCACTGTCATGAAGA			
myh6	myh6‐F	CATCCAGTACTTTGCGAGCA	718 bp	58	Feng et al. (2019)
	myh6‐R	GTACACTGACTTTGCCACTGC			

MISA software (http://pgrc.ipk‐gatersleben.de/misa/) was used to search microsatellite loci from the genomic data of *T. siluroides* (NCBI taxonomy ID: 422203). The search conditions were as follows: the length of the microsatellite repeat unit was 3–6 bp, the number of repeat units was >5, and the flanking sequence of microsatellite loci was >300 bp. Using genomic DNA from 5 individuals of each species as a template, 100 pairs of microsatellite primers were screened. The fluorescence labeling technique was used to detect microsatellite labeling polymorphism, ABI 3730XL DNA analyzer (Thermo Fisher Scientific, USA) with a GeneScan‐500Liz size standard was used for capillary electrophoresis, liz‐500 was used as reference, GeneMarker V2.2.0 software (http://www.softgenetics.com) was used for microsatellite labeling typing. Eleven pairs of highly specific and polymorphic microsatellite primers were obtained for subsequent experimental analysis (Table 3). The PCR amplification system was the same as that of the previous COI gene fragment.

**TABLE 3:** The set of microsatellite loci used in the study.

Locus	Repeat motif	Sequence of primer	Tm (°C)	Number of alleles
SSR05	(AGAA)_*n*_	F: CAGACGTGTGGGACAGATAAAGA	60	13
		R: GTTTGACGTGACATGAGGTGAAT		
SSR16	(ATG)_*n*_	F: CTAGACTGCTGGATGAGTGTCTG	60	15
		R: TTTTTGCCGACAAAGAACGTTGA		
SSR20	(ACC)_*n*_	F: AGTCTACACATACTCTTCCGTTTCA	59	9
		R: CAGTGGACTGTCATTTTCCTGAT		
SSR21	(AATAG)_*n*_	F: AAATCCGGGTTTGTAGGTCTGAA	60	4
		R: AATGGCTCCCTTGTTTTCTTTCG		
SSR26	(TGTGT)_*n*_	F: CAGGACTCGATTGACCTAGACTG	60	9
		R: CAGCCTCAAACATCTCTGTCTCT		
SSR43	(AGGTCT)_*n*_	F: CACCAGGGAGAGACTTCATACAT	60	10
		R: TACTCTGGTAGGGGGAGGTGTAG		
SSR52	(TTTG)_*n*_	F: ATACGGTTGGATGTCCTGGAAAG	60	4
		R: TCCAGTCCTTTTTGTCCTCTAGC		
SSR68	(AACAC)_*n*_	F: TGTTGCTTATGGAACACAATCCT	60	6
		R: TCTACTTATCCCCGTCATTTGGC		
SSR69	(TGT)_*n*_	F: CATGCTTCATGTCTCTCAAAGCC	60	12
		R: ACTAACCAGCAACTAACGGGAAA		
SSR76	(CCT)_*n*_	F: CCCTGTCCTCCTCTTTCTTCTTT	59	5
		R: AAGAAGAAAGGAAAAGCCAGAGC		
SSR78	(TCCA)_*n*_	F: AAGAAGAAATTTGGGGGTGCAAG	58	8
		R: TGACATTGATATGTTACTGCGGA		

### 2.3 Genetic diversity and phylogenetic analysis

CLUSTAL X 1.83 software (Thompson et al., 1997) was used to compare COI, RAG1, sRH1, and MYH6 gene sequences with default values. Haplotype number (Nh), haplotype diversity (Hd), and nucleotide diversity (π) were calculated using DnaSP 4.2 software (Rozas et al., 2003) based on the mitochondrial COI gene sequence. Using Network 4.6 software (Polzin & Daneschmand, 2003), a Media‐Joining haplotype Network map was constructed based on COI, RAG1, sRH1, and Myh6 gene sequences, and the connections between haplotypes were determined using maximum parsimony.

Using *Hedinichthys yarkandensis* as an outgroup, the phylogenetic tree was constructed by maximum likelihood (ML), Bayesian (BI), and neighbor‐joining (NJ) methods. jModelTest Version 0.1.1 software (Posada, 2008) was used to select the optimal base substitution model for each partition. ML analysis was constructed using the RAxML 8.2.10 software package (Stamatakis, 2014). To find the evolutionary tree with the highest likelihood rate, 1000 repetitions were conducted under the GTRGAMMA model, and 1000 repetitions of self‐propagation analysis were conducted to estimate the self‐propagation support of nodes. Based on the Kimura 2‐Papamter model, Mega 11.0 software (https://www.megasoftware.net/) was used to construct the phylogenetic tree using the neighbor‐joining method, and the support rate of nodes in the phylogenetic tree was estimated by 1000 self‐propagation repetitions. The Markov chain Monte Carlo (MCMC) method of MrBayes 3.2.6 software (Ronquist & Huelsenbeck, 2003) was used for Bayesian analysis. The starting tree was a random tree, and the optimal model was GTR + G. A total of 1.5 × 10^7^ generations were run, and samples were taken every 1000 generations. The first 3000 sampling trees (Burn‐in = 3000) were discarded, the remaining trees were reserved to build a majority principle consensus tree, and the Bayesian Posterior Probability (BPP) of the evolutionary tree was calculated.

To analyze phylogeny in a time‐calibrated framework, BEAST V. 2.4.6 software was used to estimate the relative time to the most recent common ancestor (Bouckaert et al., 2014). To avoid the unrealistic assumption of strict molecular clocks, we used a loose molecular clock model for analysis (Drummond et al., 2006). Two runs (10 million generations each) were performed under the GTR + G model, with parameters sampled every 1000 iterations. ESS was evaluated for the stability of posterior branching probability in Tracer 1.7 software (Rambaut et al., 2018), discarding the first 40% of the preheated evolutionary tree. The parameters and tree files obtained by the two runs were combined in LogCombiner 2.4.6 (Bouckaert et al., 2014). The maximum branch confidence tree was calculated in TreeAnnotator 2.4.6, and the final consensus tree was visualized in Figtree V.1.4 (http://tree.bio.ed.ac.uk/software/figtree/). Due to the lack of fossil evidence of the genus *Triplophysa*, this study used the mutation rate of mitochondrial genes of the genus *Cobitis* Linnaeus 1758, in which the rate of molecular evolution per million years per lineage per point was 0.68%, to estimate the differentiation time of the loach genus (Doadrio & Perdices, 2005).

### 2.4 Population history dynamic analysis

To infer population history dynamics, nucleotide mismatch analysis (Mismatch) was estimated for each species in DnaSP 4.2 software (Rozas et al., 2003). Arlequin V3.5 software was used for AMOVA and estimation of Tajima's *D* and Fu's Fs values in the neutral evolution test to judge whether the population was balanced and stable and to calculate the genetic differentiation index *F*_st_ value between each two populations (repetitions 1000) (Excoffier et al., 2007; Schneider & Excoffier, 1999).

In BEAST 2.4.6, a coalesce‐based Bayesian Skyline plot (BSP) was used to reconstruct the historical population size dynamics of each species (except *T. leptosoma*) (Drummond & Rambaut, 2007). JModelTest Version 0.1.1 software (Posada, 2008) was run twice for each species under the BIC standard, and the simulation under the MCMC method ran 50 million generations (5000, burn‐in = 25%). ESS was evaluated for the stability of posterior branching probability in Tracer 1.6 software (effective value >200), and the outputs of the two runs were combined in the LogCombiner 2.4.6 module. The BSP diagram was made in Tracer 1.6 software.

The ancestral distribution of *Triplophysa hsutschouensis* and *Triplophysa papillosolabiata* were reconstructed by RASP v4.2 (Reconstruct Ancestral State in Phylogenies, http://mnh.scu.edu.cn/soft/blog/RASP). The population was divided into three distribution areas according to water system: A: Shiyang River system; B: Heihe River system; C: Shule River system. The Bayesian final tree was used for BBM (Bayesian Binary MCMC) analysis, all parameters were kept at default values, and JC + G model was used for calculation. In the Bayesian analysis, 10 Markov chains are used to carry out two independent operations. The algebra of Markov chains is 50 million, and samples are taken every 100 generations. The first 10 million generations are discarded in the final analysis, and the results of the remaining 40 million generations are used to estimate the probability of existence or absence on each node.

### 2.5 Genetic variation and structure based on microsatellite data

All loci and groups of Hardy–Weinberg equilibrium (HWE) deviation were detected using the Markov chain Monte Carlo (MCMC) method in GENEPOPV. 3.4 (http://genepop.curtin.edu.cn). The number of alleles (A), average number of alleles (Na), effective number of alleles (Ne), observed heterozygosity (Ho), and expected heterozygosity (He) were calculated in GENETIX software (Belkhir et al., 2004). Polymorphism information content (PIC) was calculated by CERVUS 3.0 (http://www.fieldgenetics.com/pages/aboutCervus_New.jsp).

An individual‐based Bayesian clustering method was used to detect the optimal genetic structure of the population in STRUCTURE 2.3.4 (Hubisz et al., 2009). Twenty independent simulations for each *K* value ranging from 1 to 20 were run, and one million generations were run at a time, discarding the first 100,000 generations. STRUCTURE HARVESTER (Earl & Vonholdt, 2012), a web‐based software program, was used to parse and summarize the STRUCTURE output data and infer the optimal true population.

## 3 RESULTS

### 3.1 Species distribution

A total of 548 specimens of plateau loach *Triplophysa* were collected in the Qilian Mountains, and 11 species were identified. *Triplophysa shiyangensis* and *T. wuweiensis* are mainly distributed in the Shiyang River system, and *T. wuweiensis* is only distributed in the Xida River in the upper reaches of the Shiyang River. *Triplophysa strauchii* is distributed in the middle and upper reaches of the Hei River, and *T. leptosoma* is only collected in the Changma section of the upper reaches of the Shule River. *Triplophysa papillosolabiata* and *T. hsutschouensis* are widely distributed in three inland rivers (Shiyang, Hei, and Shule Rivers). However, *T. tenuis* is only distributed in the Hei and Shule Rivers, and no specimens have been collected in the Shiyang River. *Triplophysa siluroides* and *T. scleroptera* are mainly distributed in the Datong and Huangshui Rivers, the outflow rivers south of the Qilian Mountains. *Triplophysa stoliczkae* and *T. robusta* are mainly distributed in the tributaries of the Yellow River, and a few specimens were also collected in the Shiyang River system.

### 3.2 Genetic diversity

The mitochondrial COI gene dataset from the Qilian Mountains contained 557 sequences, which produced 79 haplotypes (Table 1). *T. shiyangensis* and *T. robusta* had the highest haplotype diversity (0.889 and 0.915, respectively), while *T. hsutschouensis* had the lowest haplotype diversity (0.167). The highest nucleotide diversity was found in *T. shiyangensis* (0.00636), and the lowest was found in *T. tenuis* (0.00033).

The 11 pairs of microsatellite loci showed rich genetic diversity in each species. The highest numbers of effective and observed alleles were 2.571 and 4.727 in the Changma population of *T. papillosolabiata*, respectively. The lowest value was 1 in the Datonghe River population of *T. scleroptera*. The average heterozygosity of the Danghe River population of *T. papillosolabiata* was highest (0.647), and that of the Tianzhu population of *T. scleroptera* was lowest (0.043). The average expected heterozygosity was consistent with the average observed heterozygosity. The highest value of polymorphism information content was 1.056 in the Changma population of *T. papillosolabiata*, and the lowest value was 0.148 in *T. siluroides*. The PIC of the five population of *T. hsutschouensis* in Shiyang River was the highest (0.460–0.704), followed by the Heihelinze population (0.466), and the PIC of the two population of *T. hsutschouensis* in Shule River was the lowest (0.305–0.398). The PIC of the two population of *T. papillosolabiata* in Shule River was the highest (0.948–1.056), followed by the two population in Hei River (0.850–0.941), while the Xidahe River population and Sugan lakes populations were lower (0.756 and 0.790, respectively).

### 3.3 Genetic differentiation

**FIGURE 2:**
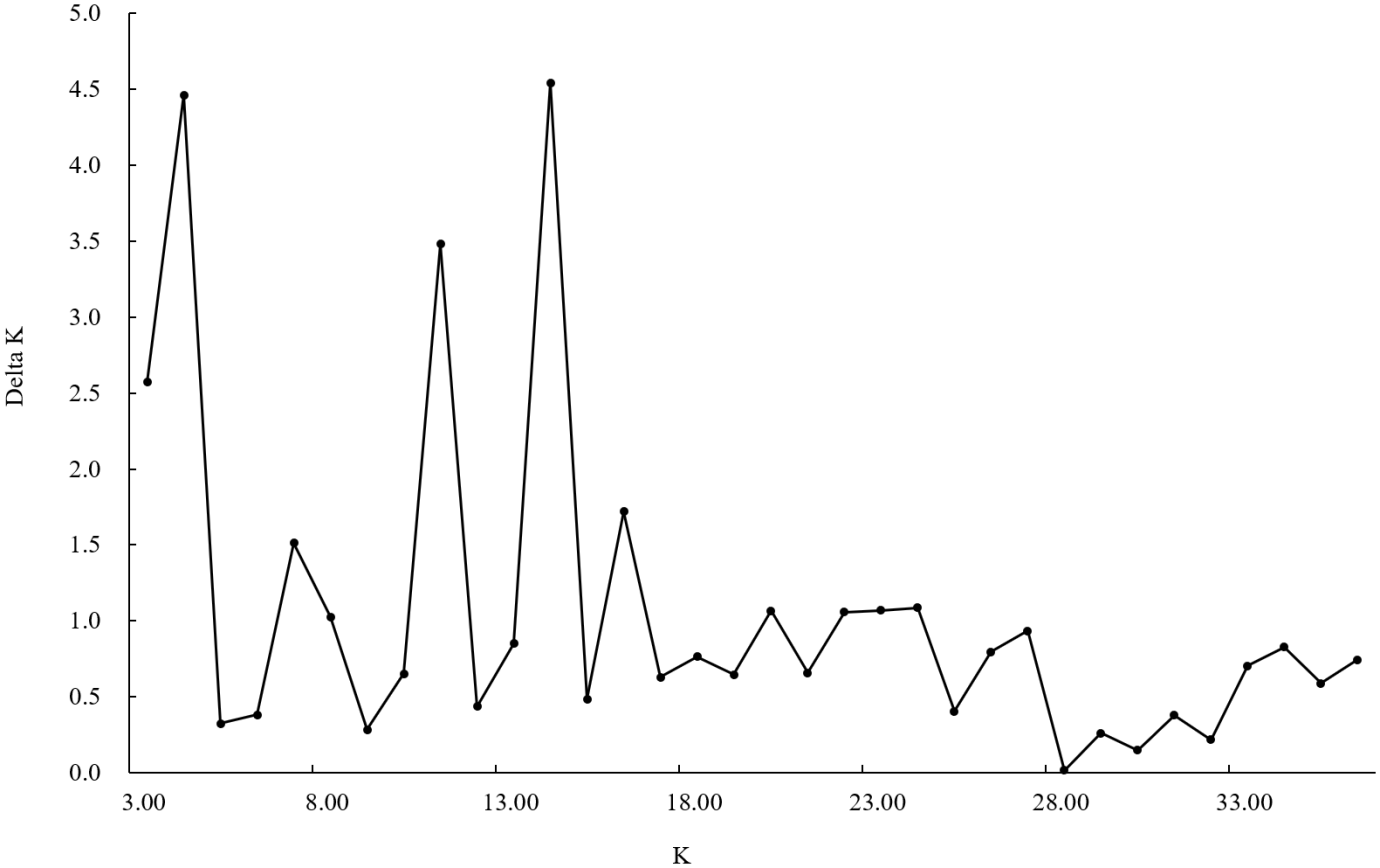
Posterior probability clustering number is calculated in STRUCTURE software based on the Bayesian method.

**FIGURE 3:**
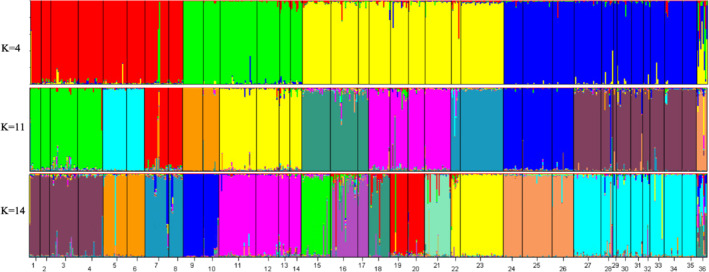
Genetic clustering graph of microsatellite data based on the Bayesian method. (1: *Triplophysa shiyangensis* Zamuhe River population; 2: *T. shiyangensis* Huangcheng reservoir population; 3: *T. shiyangensis* Huangyanghe River population; 4: *T. shiyangensis* Xidahe River population; 5: *T. stolickai* Shibalibao reservoir population; 6: *T. stolickai* Tianzhu population; 7: *T. strauchii* Heihelinze population; 8: *T. strauchii* Babaohe River population; 9: *T. scleroptera* Datonghe River population; 10: *T. scleroptera* Tianzhu population; 11: *T. tenuis* Changma population; 12: *T. tenuis* Heihelinze population; 13: *T. tenuis* Shuangta reservoir population; 14: *T. tenuis* Danghe River population; 15: *T. leptosoma* Changma population; 16: *T. papillosolabiata* Changma population; 17: *T. papillosolabiata* Danghe River population; 18: *T. papillosolabiata* Babaohe River population; 19: *T. papillosolabiata* Heihelinze population; 20: *T. papillosolabiata* Yanglong population; 21: *T. papillosolabiata* Sugan Lakes population; 22: *T. papillosolabiata* Xidahe River population; 23: *T. wuweiensis* Xidahe River population; 24: *T. siluroides* Datonghe River population; 25: *T. robusta* Shibalibao reservoir population; 26: *T. robusta* Tianzhu population; 27: *T. hsutschouensis* Changma population; 28: *T. hsutschouensis* Xidahe River population; 29: *T. hsutschouensis* Zamuhe River population; 30: *T. hsutschouensis* Heihelinze population; 31: *T. hsutschouensis* Huangcheng reservoir population; 32: *T. hsutschouensis* Jinchuanxia reservoir population; 33: *T. hsutschouensis* Shibalibao reservoir population; 34: *T. hsutschouensis* Shuangta reservoir population; 35: *T. hsutschouensis* Babaohe River population; 36: *Hedinichthys yarkandensis* Shuangta reservoir population).

**FIGURE 4:**
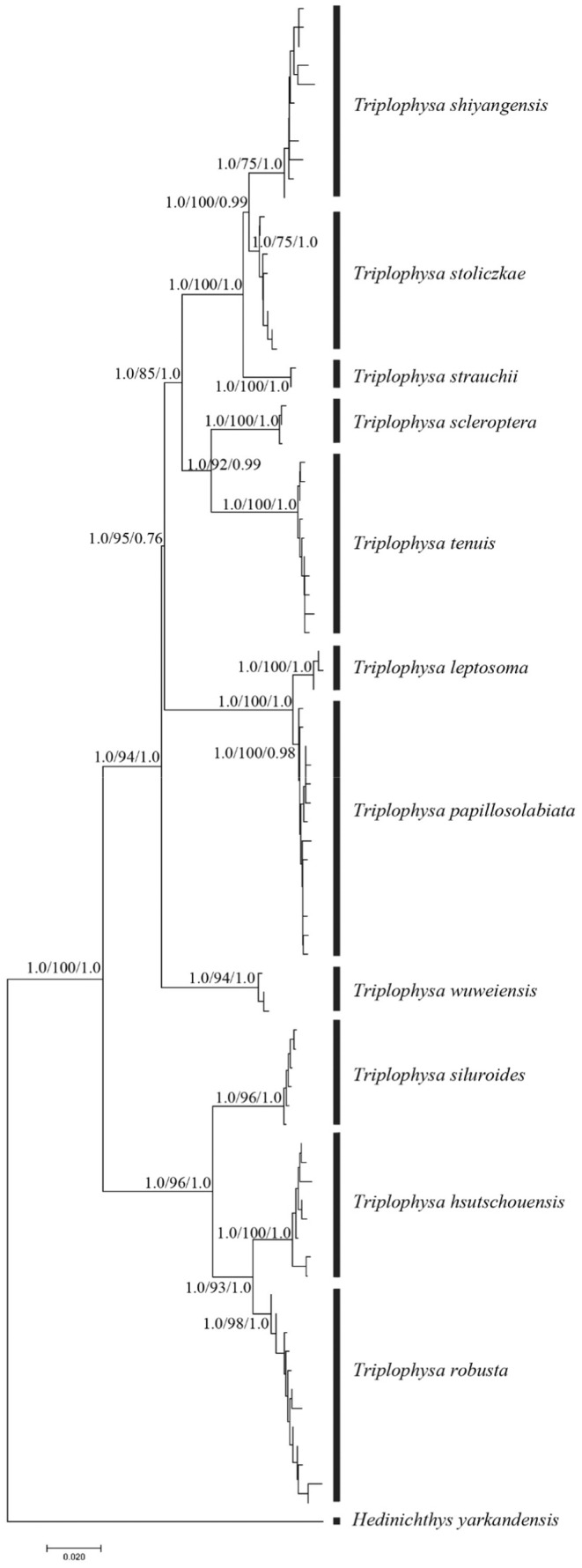
Phylogenetic tree of the *Triplophysa* fishes based on COI gene sequence. (The values at the node represent support values in NJ/ML/BI analysis, respectively, (NJ bootstrap supports values below 0.50, ML bootstrap support values below 50, and Bayesian posterior probabilities below .50 are not shown). The length of clade indicates the percentage of divergence. The ruler at the bottom represents a 0.02 replacement for each site).

The optimal structure model separated the genetic variation into 14 clusters, and the other acceptable models were 4 and 11 clusters, respectively (Figure 2). The species differentiation in the phylogenetic tree based on mitochondrial genes and nuclear genes was in good agreement with the optimal cluster shown in the structure diagram based on microsatellite data (Figures 3 and 4). When the *K* value was 14, *T. papillosolabiata* with high genetic diversity was further divided into 5 populations with obvious genetic differentiation. The first population (CSC1) included individuals from the Changma and Danghe sections of the Shule River. The second population (CSC2) consisted of samples from the Babaohe section of the upper Heihe River. The third population (CSC3) was composed of individuals from Linze section of lower reaches of the Heihe River and the Yanglong section of the Taolai River, a tributary of upper reaches of the Heihe River. The fourth (CSC4) was made up of the Suganhu population. The fifth population (CSC5) was composed of individuals from Shiyang River, but this population was divided into the sympatric distribution of *T. wuweiensis* in the optimal structure model.

The genetic differentiation index (*F*_st_) and AMOVA were analyzed based on COI gene sequences between the five populations of *T. papillosolabiata* and 10 other species (Tables 4 and 5). The results of the genetic differentiation index showed that there were no significant differences between CSC1 and CSC2, between CSC3 and CSC2, between CSC5 and CSC3, between CSC2 and CSC5, or between CSC1 and CSC5 (*p* > .01), but the statistical tests of *F*_st_ values between the other two species showed extremely significant. The *F*_st_ values between the CSC2 and CSC3 and CSC2 and CSC5 populations were negative, and the *F*_st_ values between the CSC4 and CSC2 populations were the largest (0.88387). The *F*_st_ value between the CSC2 population and *T. strauchii* was the largest (0.99470), followed by that between the CSC2 population and *T. tenuis* (0.99397). The *F*_st_ value between *T. stoliczkae* and *T. shiyangensis* was the lowest (0.86247), except for the populations of *T. papillosolabiata*. The results of AMOVA (Table 5) showed that 98.05% of the molecular differences were between species and 1.95% of the molecular differences were within species, indicating significant genetic differentiation among species (*F*_st_ = 0.98052; *p* < .001). The AMOVA (Table 6) of the five populations of *T. papillosolabiata* showed that 82.06% of the molecular differences were within the populations, and 17.94% of the molecular differences were between the populations. The genetic differentiation among the populations was also extremely significant (*F*_st_ = 0.17944; *p* < .001). The genetic differentiation index analysis based on nuclear genes between species revealed significant genetic differentiation between 11 species pairs, except that *T. leptosoma* and *T. papillosolabiata* shared the same haplotype based on sRH1 and Myh6 gene sequences (Table 7).

**TABLE 4:** Pairwise *F*_st_ (below diagonal) and associated *p* values (above diagonal) among the species of *Triplophysa* based on COI gene.

	1	2	3	4	5	6	7	8	9	10	11	12	13	14	15
1. *T. papillosolabiata* CSC1		0.07207	0.00000	0.00901	0.01802	0.00000	0.00000	0.00000	0.00000	0.00000	0.00000	0.00000	0.00000	0.00000	0.00000
2. *T. papillosolabiata* CSC2	0.15357		0.77477	0.00000	0.99099	0.00000	0.00000	0.00000	0.00000	0.00000	0.00000	0.00000	0.00000	0.00000	0.00000
3. *T. papillosolabiata* CSC3	0.20008	−0.03076		0.00000	0.36937	0.00000	0.00000	0.00000	0.00000	0.00000	0.00000	0.00000	0.00000	0.00000	0.00000
4. *T. papillosolabiata* CSC4	0.45427	0.88387	0.79699		0.00000	0.00000	0.00000	0.00000	0.00000	0.00000	0.00000	0.00000	0.00000	0.00000	0.00000
5. *T. papillosolabiata* CSC5	0.18628	−0.04046	0.01075	0.84491		0.00000	0.00000	0.00000	0.00000	0.00000	0.00000	0.00000	0.00000	0.00000	0.00000
6. *T. tenuis*	0.99100	0.99397	0.99333	0.99354	0.99382		0.00000	0.00000	0.00000	0.00000	0.00000	0.00000	0.00000	0.00000	0.00000
7. *T. hsutschouensis*	0.97484	0.97158	0.97529	0.96851	0.97363	0.98439		0.00000	0.00000	0.00000	0.00000	0.00000	0.00000	0.00000	0.00000
8. *T. siluroides*	0.98499	0.98793	0.98882	0.98428	0.98884	0.99283	0.93495		0.00000	0.00000	0.00000	0.00000	0.00000	0.00000	0.00000
9. *T. stoliczkae*	0.97140	0.97123	0.97542	0.96503	0.97401	0.98370	0.97007	0.97848		0.00000	0.00000	0.00000	0.00000	0.00000	0.00000
10. *T. leptosoma*	0.86703	0.95209	0.92516	0.90985	0.94252	0.99333	0.97061	0.98532	0.96862		0.00000	0.00000	0.00000	0.00000	0.00000
11. *T. shiyangensis*	0.97342	0.97207	0.97678	0.96576	0.97506	0.98585	0.97044	0.97894	0.86247	0.96938		0.00000	0.00000	0.00000	0.00000
12. *T. wuweiensis*	0.98250	0.99301	0.99032	0.98879	0.99222	0.99182	0.96726	0.98261	0.97166	0.98944	0.96908		0.00000	0.00000	0.00000
13. *T. strauchii*	0.98427	0.99470	0.99179	0.99032	0.99372	0.99133	0.97261	0.98745	0.91190	0.99078	0.93224	0.98991		0.00000	0.00000
14. *T. scleroptera*	0.98315	0.99259	0.99041	0.98765	0.99206	0.98754	0.96998	0.98506	0.96315	0.98832	0.96536	0.98683	0.98778		0.00000
15. *T. robusta*	0.97984	0.97835	0.98178	0.97470	0.98047	0.98945	0.87205	0.95282	0.97456	0.97631	0.97434	0.97573	0.97994	0.97732	

**TABLE 5:** Analysis of molecular variance (AMOVA) among the species of *Triplophysa* fishes based on COI gene.

Source of variation	d.f.	Sum of squares	Variance components	Percentage of variation	Fixation index	Significance
Among species	14	10971.708	28.39652 Va	98.05	0.98052	*p* < .001
Within species	534	239.766	0.56416 Vb	1.95		
Total	548	11211.474	28.96067			

**TABLE 6:** Analysis of molecular variance (AMOVA) among the *Triplophysa papillosolabiata* based on COI gene.

Source of variation	d.f.	Sum of squares	Variance components	Percentage of variation	Fixation index	Significance
Among populations	4	3.591	0.04277 Va	17.94	0.17944	*p <* .001
Within populations	105	17.017	0.19560 Vb	82.06		
Total	109	20.609	0.23837			

**TABLE 7:** *F*_st_ values between different species of the *Triplophysa fishes* based on three nuclear genes RAG, sRH1, and myh6.

	1	2	3	4	5	6	7	8	9	10
*RAG*										
1. *T. papillosolabiata*										
2. *T. tenuis*	0.92552									
3. *T. hsutschouensis*	0.98006	0.96610								
4. *T. siluroides*	0.96287	0.94964	0.91437							
5. *T. stoliczkae*	0.91510	0.88080	0.95449	0.93781						
6. *T. leptosoma*	0.88539	0.92928	0.98600	0.96625	0.91287					
7. *T. shiyangensis*	0.96825	0.94172	0.98767	0.97178	0.83507	0.97303				
8. *T. wuweiensis*	0.97256	0.90555	0.99493	0.97694	0.92494	0.98179	0.98466			
9. *T. strauchii*	0.97287	0.94660	0.99214	0.97482	0.61164	0.97925	0.95734	0.99141		
10. *T. scleroptera*	0.96948	0.88211	0.99510	0.97379	0.91698	0.98021	0.98441	0.99587	0.99147	
11. *T. robusta*	0.96436	0.95205	0.89777	0.83689	0.93930	0.96849	0.97490	0.98019	0.97788	0.97749
*RH1*										
1. *T. papillosolabiata*										
2. *T. tenuis*	0.96841									
3. *T. hsutschouensis*	0.99904	0.97635								
4. *T. siluroides*	0.99609	0.97412	0.97447							
5. *T. stoliczkae*	0.99683	0.91876	0.99626	0.99271						
6. *T. leptosoma*	0.00000	0.96841	0.99904	0.99609	0.99683					
7. *T. shiyangensis*	1.00000	0.99562	0.99971	0.99879	0.99943	1.00000				
8. *T. wuweiensis*	0.98639	0.78144	0.98895	0.98568	0.96174	0.98639	0.99809			
9. *T. strauchii*	1.00000	0.91416	0.99869	0.99479	0.94286	1.00000	1.00000	0.96503		
10. *T. scleroptera*	0.98510	0.85372	0.98810	0.98484	0.96743	0.98510	0.99798	0.91773	0.97080	
11. *T. robusta*	0.99355	0.96662	0.93165	0.90308	0.98886	0.99355	0.99816	0.98025	0.99107	0.97914
*myh6*										
*1. T. papillosolabiata*										
2. *T. tenuis*	0.97351									
3. *T. hsutschouensis*	1.00000	0.98306								
4. *T. siluroides*	0.98547	0.96105	0.9037							
5. *T. stoliczkae*	0.95175	0.88369	0.97054	0.95074						
6. *T. leptosoma*	0.00000	0.97351	1.00000	0.98547	0.95175					
7. *T. shiyangensis*	0.98766	0.91427	0.99217	0.9704	0.90496	0.98766				
8. *T. wuweiensis*	1.00000	0.95562	1.00000	0.98323	0.94691	1.00000	0.98363			
9. *T. strauchii*	0.98745	0.92745	0.99237	0.97322	0.82557	0.98745	0.95923	0.98618		
10. *T. scleroptera*	0.99731	0.89302	0.9983	0.97814	0.92925	0.99731	0.97031	0.99595	0.97845	
11. *T. robusta*	0.98897	0.96716	0.93368	0.65637	0.95604	0.98897	0.97696	0.98759	0.97803	0.98353

### 3.4 Phylogeny and haplotype network evolution

Based on the COI gene sequence, a phylogenetic tree with consistent topological structure was obtained by using maximum likelihood (ML), Bayesian inference (BI), and neighbor‐joining (NJ) methods, but the support values of some nodes were different. Therefore, only the NJ phylogenetic tree is given in this study (Figure 4). The phylogenetic tree shows that 11 plateau loach species in the Qilian Mountains possess unique haplotypes. In the topological structure of the phylogenetic tree, a single clade with high support was formed according to morphological species classification. Two clades were clearly identified from the phylogenetic tree. One clade was composed of three species, that is, *T. robusta*, *T. hsutschouensis*, and *T. siluroides*. The other clade was composed of eight species of plateau loach, that is, *T. shiyangensis*, *T. stoliczkae*, *T. strauchii*, *T. scleroptera*, *T. tenuis*, *T. leptosome*, *T.wuweiensis*, and *T. papillosolabiata*.

**FIGURE 5:**
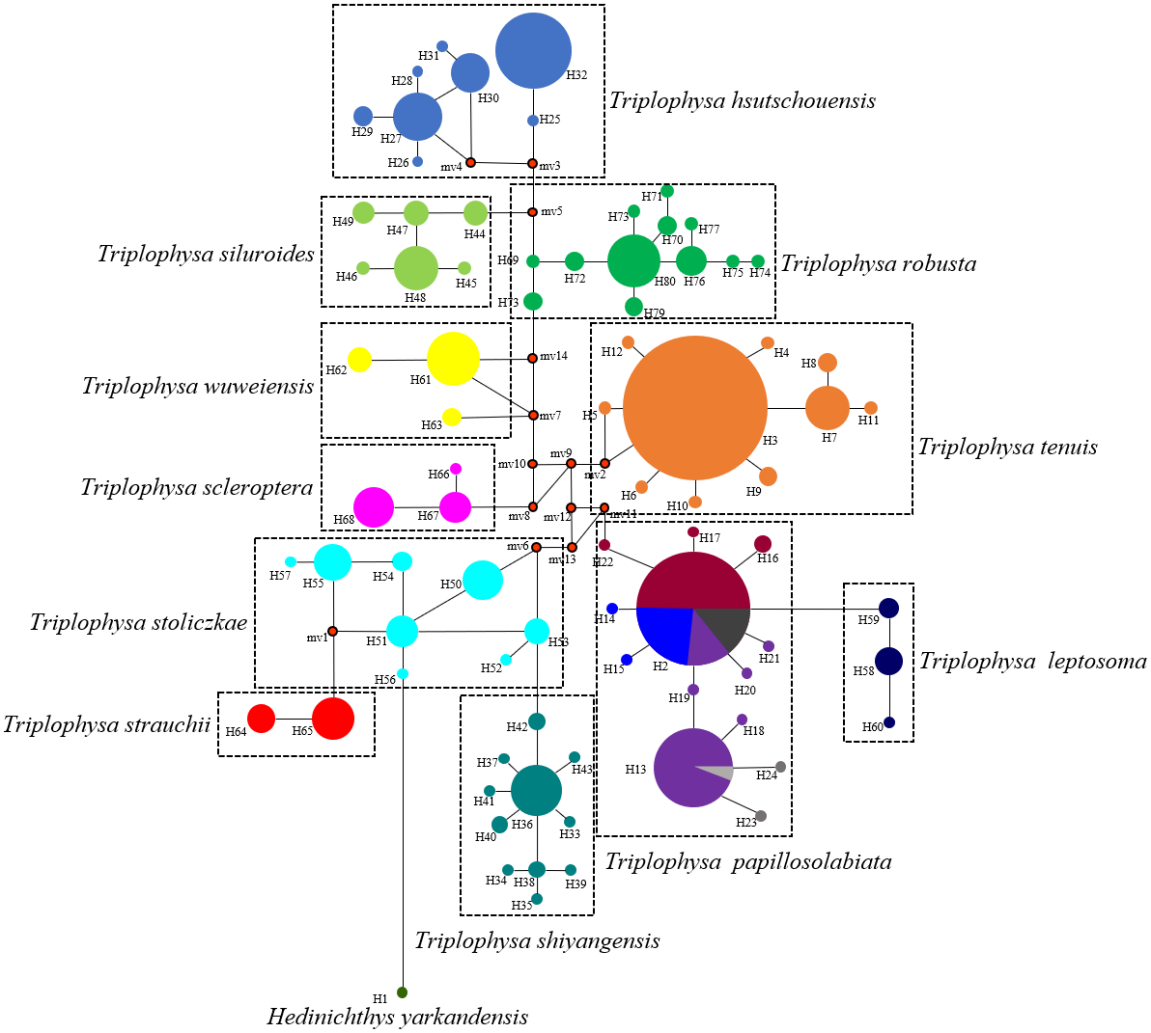
Map of minimal evolutionary network of haplotypes in the *Triplophysa* fishes. (The area of circles is proportional to the haplotype frequencies, and mv1–mv14 are missing haplotypes. Lines linking haplotypes indicate the evolutionary paths among haplotypes, vertical bars on the linking lines represent mutation steps between haplotypes).

**FIGURE 6:**
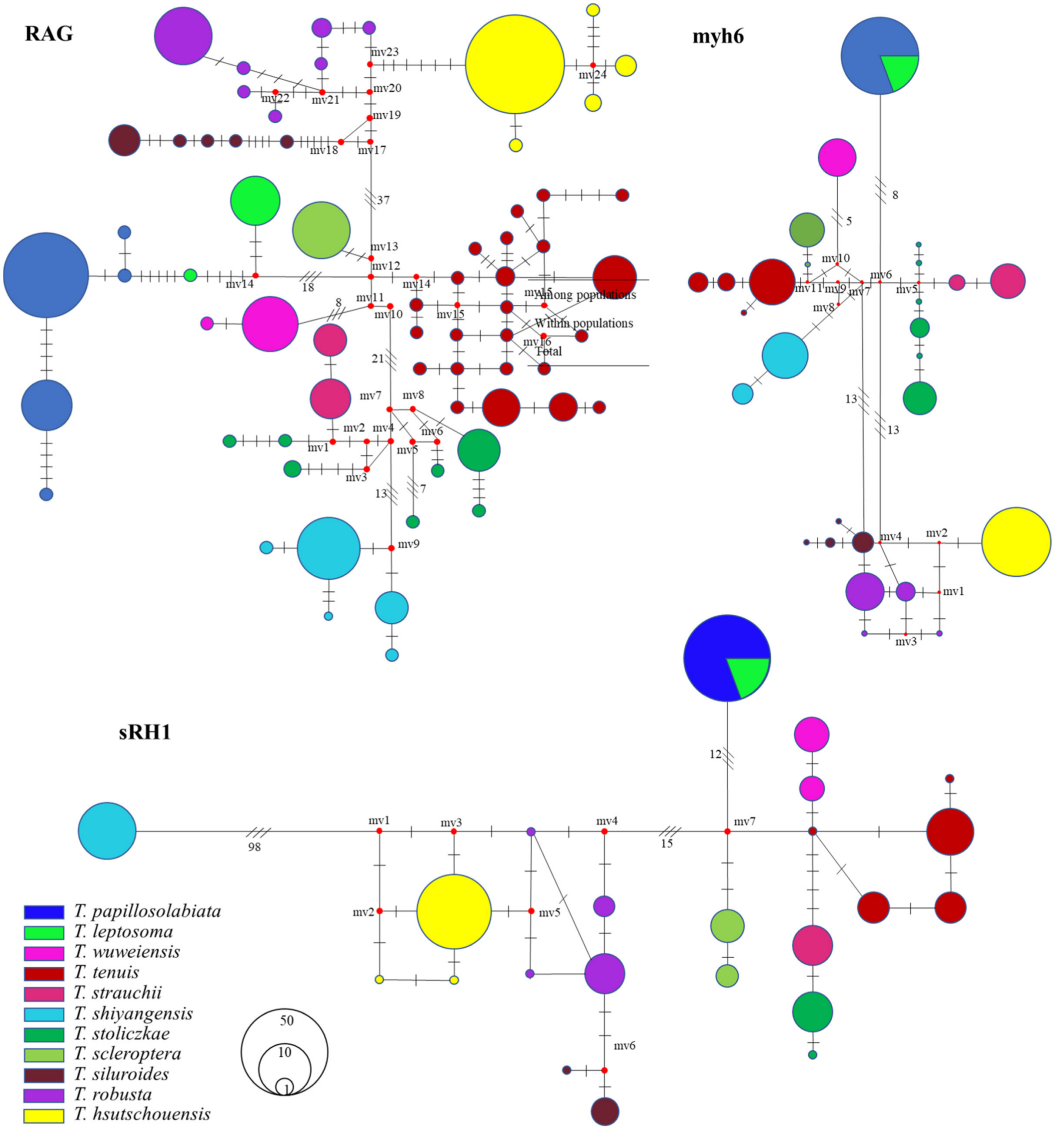
Network haplotype evolution based on three nuclear genes. (The area of circles is proportional to the haplotype frequencies, and mv1–mv24 are missing haplotypes. Lines linking haplotypes indicate the evolutionary paths among haplotypes, vertical bars on the linking lines represent mutation steps between haplotypes).

The haplotype evolutionary network diagram constructed based on the COI gene sequences clearly shows the haplotype relationships of 11 plateau loach species (Figure 5). The two major clades were connected by 1–3 steps of mutation, and there was no haplotype shared among all species. The haplotype evolutionary network diagram further supports the phylogenetic tree. The haplotype network evolution map was constructed based on the nuclear gene sequences (Figure 6), and 64, 23, and 27 alleles were obtained for RAG1, sRH1, and Myh6, respectively. The haplotype network evolution map constructed by the three nuclear genes was slightly different. In the haplotype network evolution map constructed by the RAG1 gene sequence, there were no shared alleles among the 11 plateau loach species. However, an evolutionary map of the haplotype network based on sRH1 and Myh6 gene sequences showed that both *T. leptosoma* and *T. papillosolabiata* shared a single allele.

### 3.5 Population history dynamics and species differentiation time

**FIGURE 7:**
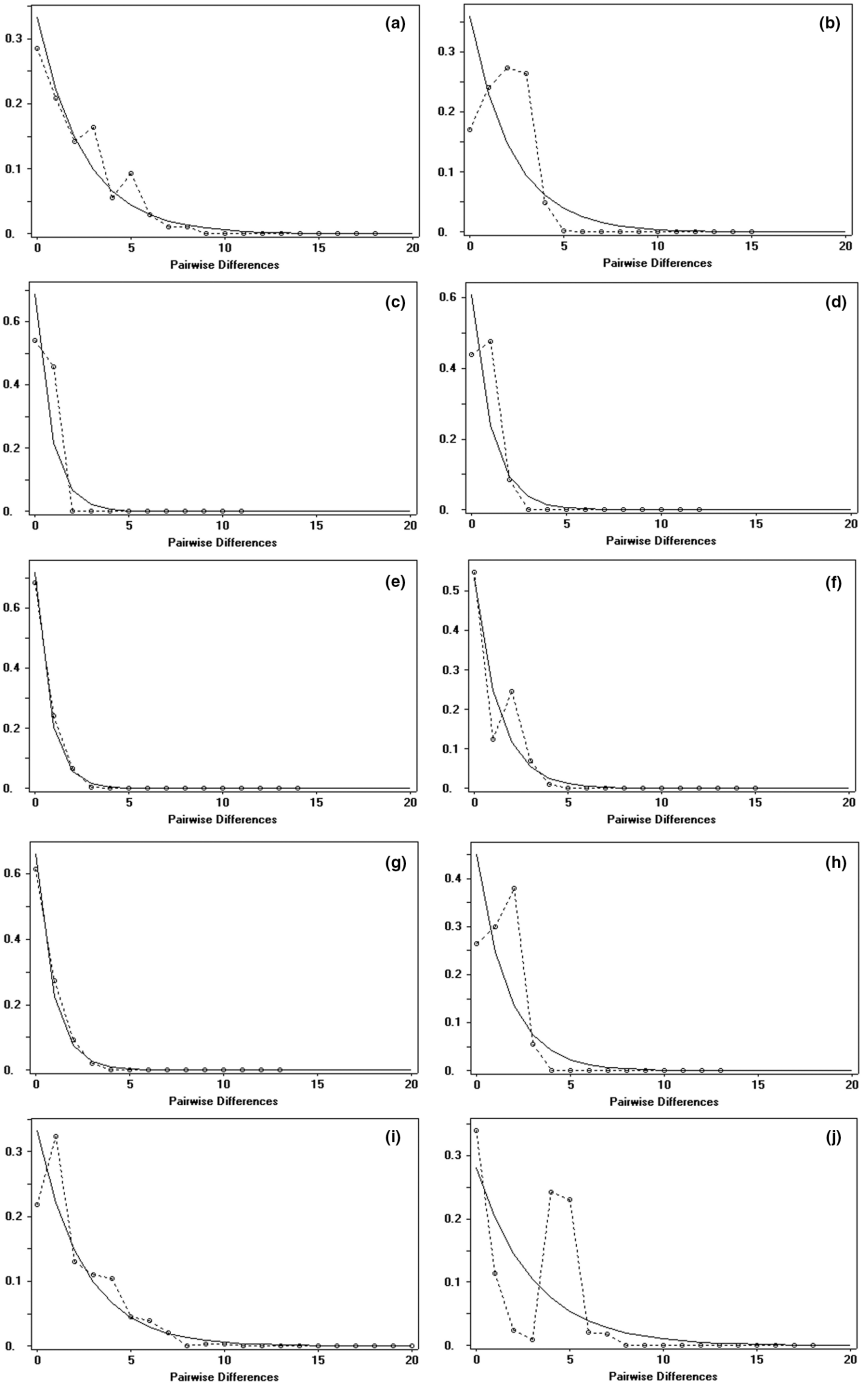
Nucleotide mismatch distribution of the *Triplophysa* fishes. (The *X*‐axis is pairwise differences and the *Y*‐axis is frequency. The solid line is the expected mismatched distribution frequency, and the dashed line is the observed mismatched distribution frequency. (a) *Triplophysa shiyangensis*; (b) *T. stoliczkae*; (c) *T. strauchii*; (d) *T. scleroptera*; (e) *T. tenuis*; (f) *T. papillosolabiata*; (g) *T. wuweiensis*; (h) *T. siluroides*; (i) *T. robusta*; (j) *T. hsutschouensis*).

**FIGURE 8:**
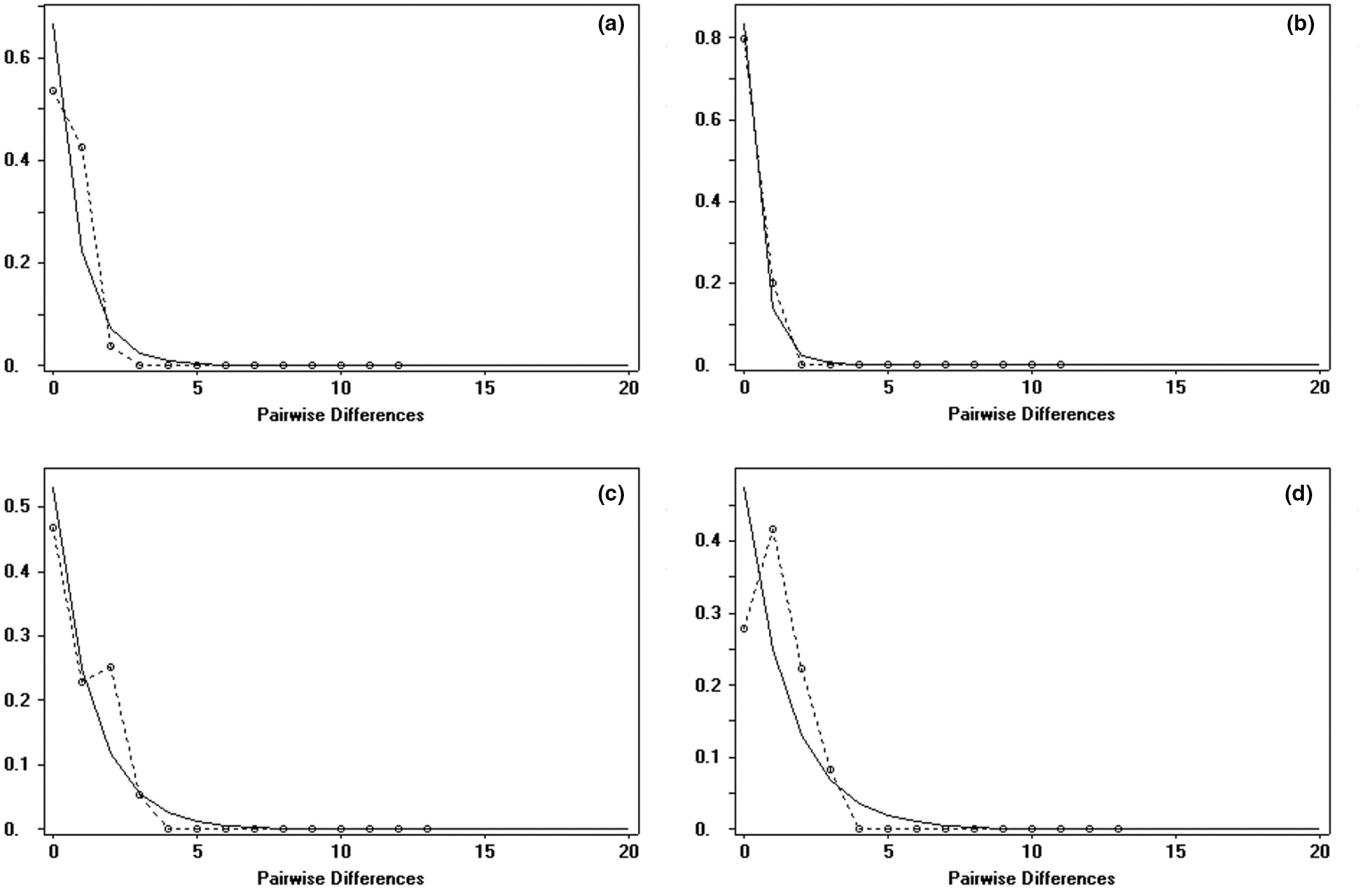
Nucleotide mismatch distribution of the *Triplophysa tenuis*. (The *X*‐axis is pairwise differences and the *Y*‐axis is frequency. The solid line is the expected mismatched distribution frequency, and the dashed line is the observed mismatched distribution frequency. (a) *Triplophysa tenuis* Changma population; (b) *Triplophysa tenuis* Danghe River population; (c) *Triplophysa tenuis* Heihelinze population; (d) *Triplophysa tenuis* Shuangta reservoir population).

**FIGURE 9:**
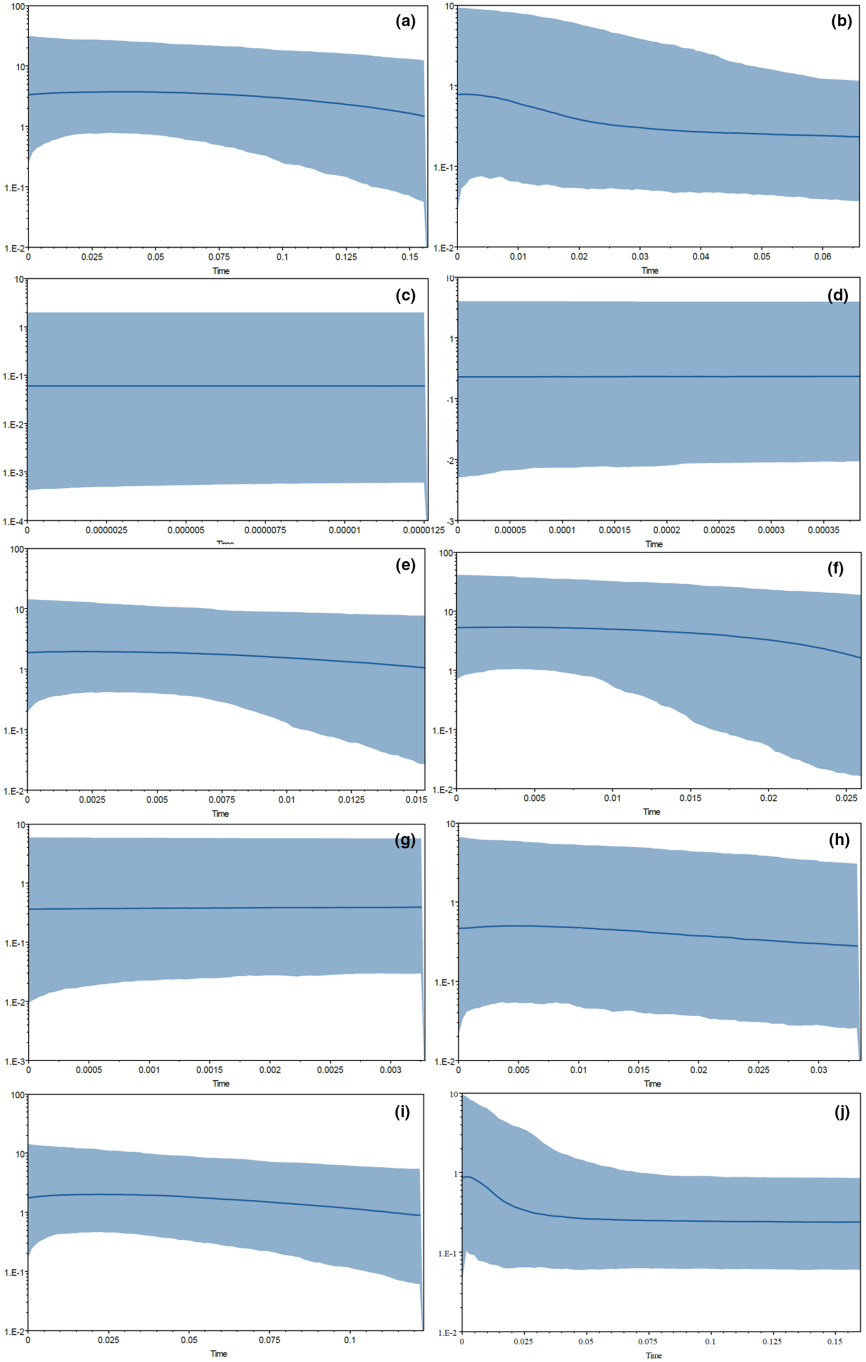
Bayesian skyline plots (BSPs) analysis of the *Triplophysa* fishes. (The *X*‐axis is million years ago and the *Y*‐axis is effective population size. The blue shaded areas represent 95% confidence intervals, and the blue lines represent median value. (a) *Triplophysa shiyangensis*; (b) *T. stoliczkae*; (c) *T. strauchii*; (d) *T. scleroptera*; (e) *T. tenuis*; (f) *T. papillosolabiata*; (g): *T. wuweiensis*; (h) *T. siluroides*; (i) *T. robusta*; (j) *T. hsutschouensis*).

The analysis of population dynamics provides an important perspective for the evolutionary history of loach species in the Qilian Mountains (Figure 7 and Table 8). The nucleotide mismatch distribution curves of *T. tenuis*, *T. siluroides*, *T. stoliczkae*, *T. wuweiensis*, *T. strauchii*, *T. scleroptera*, and *T. leptosoma* showed a single peak. The Tajima's *D* and Fu's FS tests of the 6 species of loach were not significant (*p* > .05), but those of *T. tenuis* were extremely significant (*p* < .05). The inconsistent results of this analysis may be due to the small number of haplotypes in the six species, and the small number of nucleotide variation sites may lead to the false appearance of a single peak in the analysis of nucleotide mismatch distribution. These results indicated that the six species, except *T. tenuis*, did not deviate significantly from the population expansion model. The nucleotide mismatch analysis of different geographic populations of *T. tenuis* showed a single peak only in Changma population and Danghe river population, but the Tajima' *D* and Fu's FS tests were not significant (*p* > .10), indicating that all populations of *T. tenuis* did not deviate significantly from the population expansion model (Figure 8). The nucleotide mismatch distribution of *T. hsutschouensis*, *T. shiyangensis*, *T. papillosolabiata*, and *T. robusta* showed a multipeak distribution. Tajima's and Fu's FS tests of the three species except *T. shiyangensis* were not significant (*p* > .05), and no population expansion events were detected. In contrast, the BSP diagram of each species showed that only *T. hsutschouensis* had experienced a population expansion event since approximately 0.025 MYA, while the population expansion event of *T. tenuis* was not obvious. Other species have maintained stable population sizes in recent history without significant population expansion (Figure 9).

**TABLE 8:** The Neutrality test of the *Triplophysa* fishes.

Species	Tajima's *D*	*p*	Fu's Fs	*p*
*T. papillosolabiata* CSC1	−1.49796	.1 > *p* > .05	−1.615	<.05*
*T. papillosolabiata* CSC2	−1.51177	.1 > *p* > .05	−1.519	<.05*
*T. papillosolabiata* CSC3	−0.27266	> .10	−1.162	>.10
*T. papillosolabiata* CSC4	0	1	0	N.A.
*T. papillosolabiata* CSC5	−0.70990	>.10	−0.887	>.10
*T. tenuis*	−1.96857	<.05*	−9.627	>.10
*T. tenuis* (Changma population)	0.01053	>.10	0.097	>.10
*T. tenuis* (Heihelinze population)	−1.17758	>.10	−1.186	>.10
*T. tenuis* (Shuangta reservoir population)	−1.67754	.1 > *p* > .05	−2.231	.1 > *p* > .05
*T. tenuis* (Danghe River population)	−1.11173	>.10	−0.339	>.10
*T. hsutschouensis*	−0.01370	>.10	0.745	>.10
*T. siluroides*	−0.28026	>.10	−1.427	>.10
*T. stoliczkae*	0.03222	>.10	−1.682	>.10
*T. leptosoma*	−0.27492	>.10	−0.438	>.10
*T. shiyangensis*	−1.87685	<.05*	−4.504	.1 > *p* > .05
*T. wuweiensis*	−1.13127	>.10	−0.23	>.10
*T. strauchii*	1.03439	>.10	1.096	>.10
*T. scleroptera*	0.13916	>.10	0.050	>.10
*T. robusta*	−1.62933	.1 > *p* > .05	−5.058	>.10

*Note:* “*” means significant difference.

**FIGURE 10:**
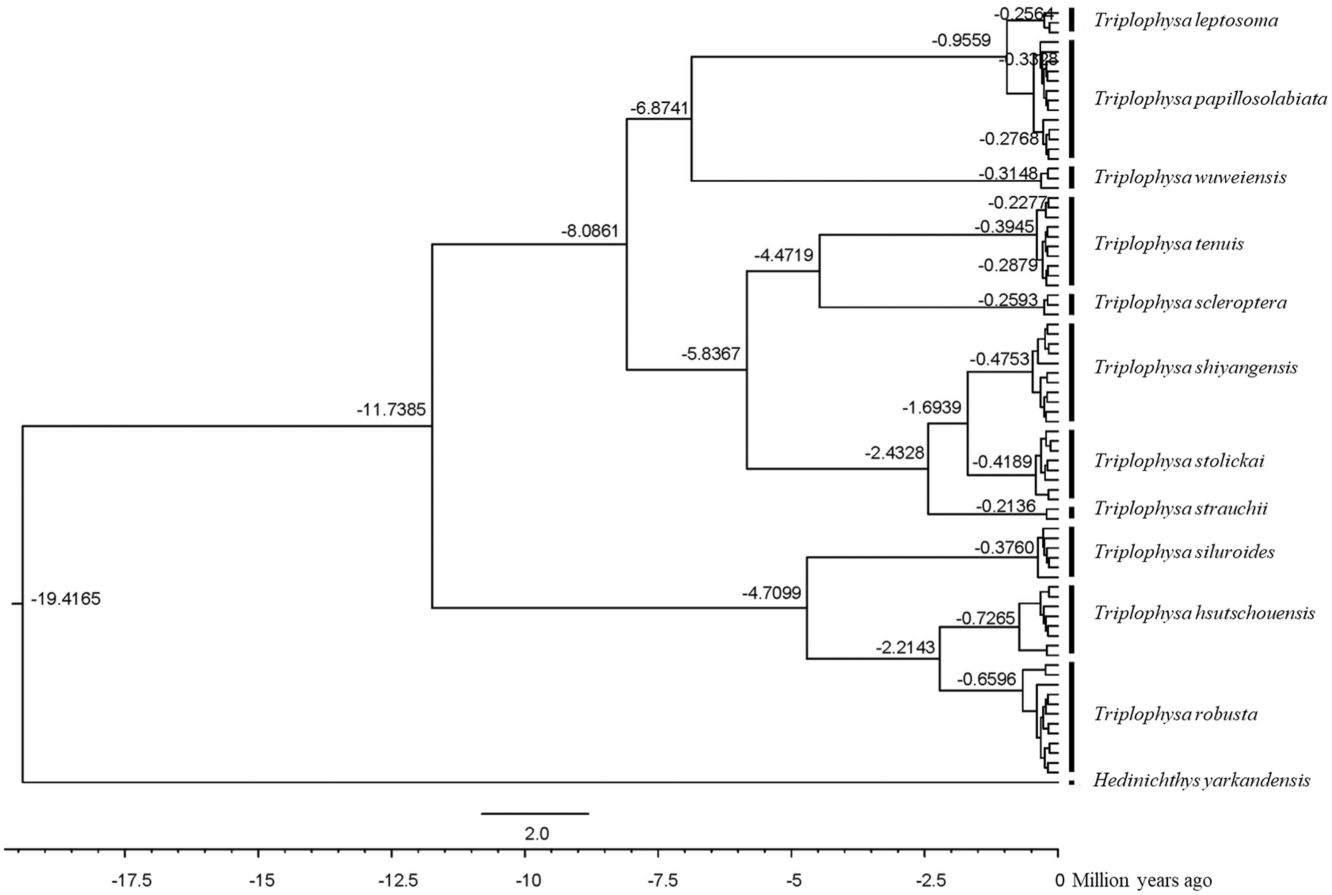
Species differentiation time of the *Triplophysa* fishes. (The lower ruler is based on BEAST differentiation time; The number at the node indicates the differentiation time).

Based on the mitochondrial gene evolution rate of 0.68% per million years, it is estimated that the time from the progenies to the most recent common ancestor of the plateau loaches in the Qilian Mountains is between 0.9559 and 19.4165 MYA (Figure 10). The time of differentiation between the genera *Triplophysa* and *Hedinichthys* was 19.4165 MYA, and the time of differentiation between the two major clades was 11.7385 MYA. Estimates of the time from progenies to common ancestors for most species range from 0.9559 MYA to 6.8741 MYA. The estimated time between *T. leptosoma* and *T. papillosolabiata* and their most recent common ancestor was 0.9559 MYA (approximately the Early Pleistocene), and the maximum estimated time to the most recent common ancestor of *T. wuweiensis*, *T. leptosoma*, and *T. papillosolabiata* was 6.8741 MYA (approximately the Late Pliocene). The estimation of the time from the earliest to the most recent common ancestor of loach species in the Qilian Mountains suggests that species differentiation occurred in the Late Pliocene and early Pleistocene.

**FIGURE 11:**
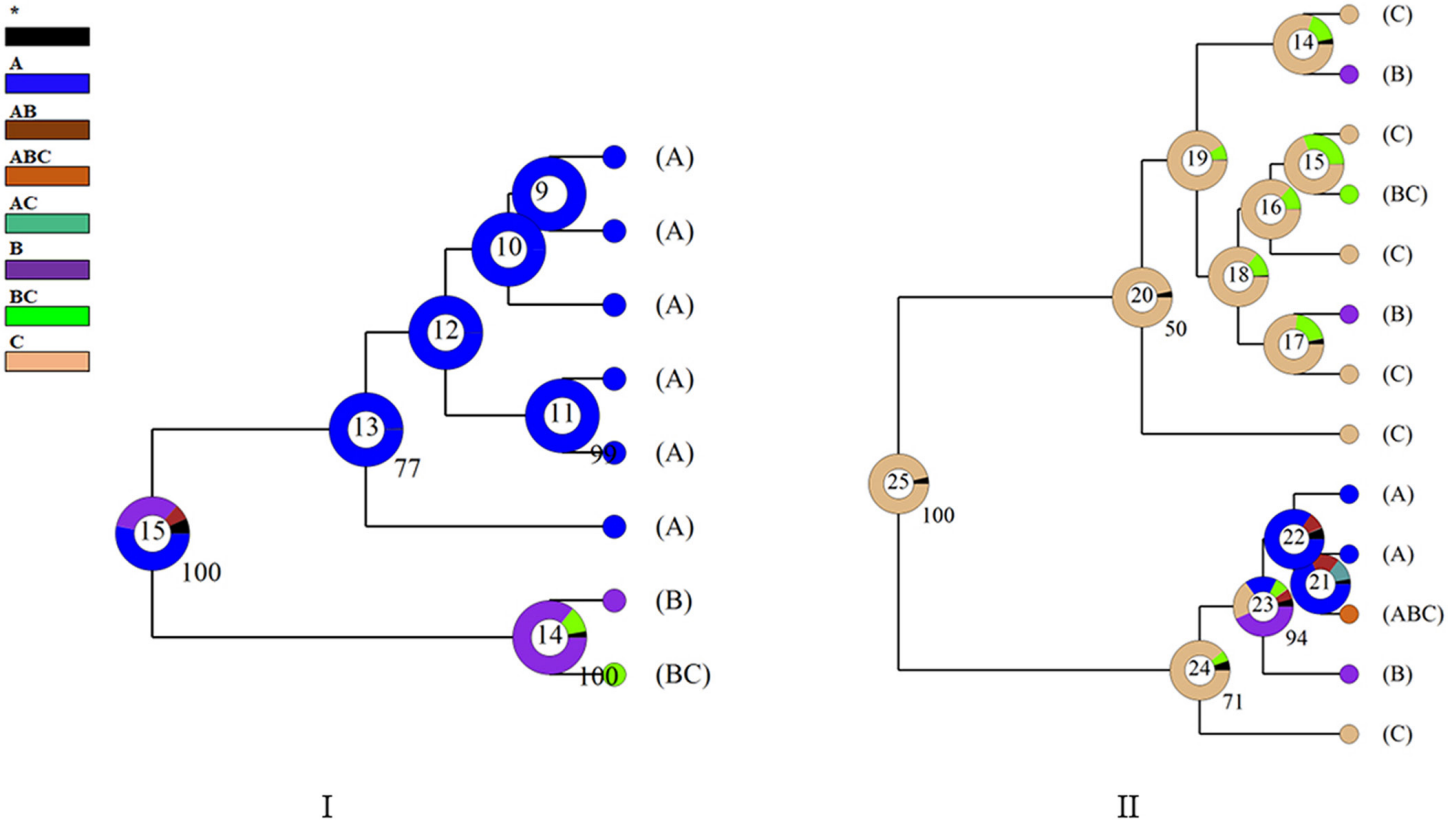
Reconstructed ancestral distribution of *Triplophysa hsutschouensis* (I) and *Triplophysa papillosolabiata* (II) based on BBM. (a: Shiyang River system; b: Heihe River system; c: Shule River system; * represent other ancestral ranges. The different colors of the nodes represent different ancestral ranges. The number in the ring represents the node's coded. The values at the node represent support values in Bayesian analysis, and the posterior probability of less than 50 is not shown).

The results of the reconstruction of the ancestral distribution region of *Triplophysa hsutschouensis* based on BBM showed that the ancestral distribution region at node 15 included several possible distribution regions, among which the proportion of ancestral distribution area of Shiyang River system is dominant (Figure 11). The results of BBM analysis on the population of *Triplophysa papillosolabiata* showed that the distribution type of the Shule River system was the ancestral distribution area of *T. papillosolabiata*.in the Qilian Mountains.

## 4 DISCUSSION

### 4.1 Species distribution and colonization

There has been no detailed investigation of the species distribution of plateau loach in the Qilian Mountains, and only a few sporadic investigations have been carried out. Some new endemic species of plateau loach have been discovered successively (Li & Chang, 1974; Zhao, 1984; Zhu & Wu, 1981), but the precise range of species remains to be revealed. In this study, based on an extensive specimen collection, we present a detailed distribution of all 11 species of plateau loach in the Qilian Mountains. *Triplophysa siluroides* and *T. scleroptera* are distributed in the Datong River and Huangshui River tributaries of the Yellow River in the southern Qilian Mountains. *Triplophysa robusta* and *T. stoliczkae* are mainly distributed in the Huangshui River but also exist in the Gulang River, a tributary of the Shiyang River, which may be the remains of the Wuwei Basin after its isolation from the Yellow River, corresponding to their low genetic diversity (Feng, 1963). When the species are distributed discretely or mixed with discrete and continuous distribution, the marginal population is in a relatively isolated state, and it is easy to produce a small population completely separated from the main population. Such populations can evolve into new species over time (Coyne & Orr, 2004). *Triplophysa wuweiensis* is only distributed in the Xida River in the west of Shiyang River, and is considered to be a new species formed in the Miocene due to the geographical isolation between the Wuwei Basin and the Yellow River system (Li & Chang, 1974). *Triplophysa shiyangensis* is distributed only in the Shiyang River system and is considered a specialized species independently adapted to the water environment of the Shiyang River (Zhao & Wang, 1983).

*Triplophysa hsutschouensis* is widely distributed in the Shiyang, Heihe, and Shule Rivers, and is regarded as an effective species because its body length is 7.18–7.60 times of body height. Wang (1991) believed that the uplift of the Wushaoling Mountains led to the isolation of the inland River of the Hexi Corridor and the Yellow River. *T. hsutschouensis* was differentiated from *T. robusta* due to geographical isolation, but the geographical isolation time was quite different from the species differentiation time of *T. hsutschouensis* and *T. robusta* in this study (2.2143 MYA). The main morphological difference between *T. hsutschouensis* and *T. robusta* lies in the different degrees of body surface scales. The body surface of *T. hsutschouensis* is almost bare and scaleless, which can reduce the waste of body temperature in cold areas (Wang, Yang, et al., 2015; Wang, Zhang, et al., 2015; Wang et al., 2016; Yang et al., 2021). This may be due to scale degradation during the Pleistocene Ice Age, which was an adaptive evolution of *T. hsutschouensis* to a cold environment. Different landscape features and climate patterns can drive directional selection (Sobel et al., 2010), which may play an important role in the speciation process of *T. hsutschouensis*. The process of population expansion is often accompanied by drastic population genetic changes. The genetic diversity of this marginal population decreased due to bottleneck effect or genetic drift (Gao & Gao, 2016; Hirsch et al., 2015; Yang et al., 2016). According to the genetic diversity of geographical populations in different water systems (Table 1), it is obvious that the polymorphic information index based on microsatellite markers of the five populations of *T. hsutschouensis* in the Shiyang River system was the highest (0.460–0.704), followed by the Heihelinze populations in the Hei River (0.466) and the two populations in Shule River (0.305–0.398). The genetic diversity of different geographical populations of *T. hsutschouensis* showed an obvious decreasing trend from the Shiyang River to the Hei and Shule Rivers. In the analysis of mitochondrial gene markers, although some populations have only one haplotype or a small population can reduce the genetic diversity of the population (Wang et al., 2017; Zhang et al., 2014), this trend of genetic diversity reduction can still be found. The population of *T. hsutschouensis* began to colonize from east to west along the northern Qilian Mountains. A population expansion event occurred at approximately 0.025 MYA during the last glacial period (0.075–0.01 MYA) on the Tibetan Plateau. This is consistent with the dispersal pattern of the sympatric species *Schizopygopsis chilianensis* (Zhao et al., 2011).

*Triplophysa tenuis*, *T. strauchii*, and *T. papillosolabiata* are mainly distributed in the Tarim Basin (Wu & Wu, 1992). The inland river system of the Hexi Corridor is the eastern edge of the species distribution area. *T. tenuis* was mainly distributed in the Shule and Heihe Rivers, while *T. strauchii* was only collected in the Linze section of the middle reaches of the Hei River but not in the Shule River. This phenomenon of a discontinuous distribution area may be due to the influence of the ice age, and the Hei population of *T. strauchii* could take refuge in the lower reaches of the Hei River at low altitudes so that the population could survive. However, the lower reaches of the Shule River had high altitudes and could not form a shelter, leading to the extinction of the Shule River population. This phenomenon also exists in the species distribution of *Rana chensinensis* (Zhou et al., 2012). *Triplophysa papillosolabiata* is widely distributed in all water systems of the Hexi Corridor, which may be related to its strong ecological adaptation. It can not only feed in the rapids of the Dang River at an elevation of 2300 m but also survive in trickle water and even survive in some small water bodies frozen to the bottom in winter (Zhao, 1984). According to geographical distribution and genetic diversity, the PIC of the two population of *T. papillosolabiata* in Shule River was the highest (0.948–1.056), followed by the two population in Hei River (0.850–0.941), while the Xidahe River population and Sugan lakes populations were lower (0.756 and 0.790, respectively). The genetic diversity of populations in different river systems showed an obvious decreasing trend from Shule River to Hei River and Shiyang River. *T. papillosolabiata* colonized from west to east along the northern Qilian Mountains from the Shule River as a starting point. *Triplophysa leptosoma* was only collected in the Changma section of the upper reaches of the Shule River. Combined with the data of nuclear and mitochondrial genes, *T. leptosoma* was considered to be a new species that differentiated from *T. papillosolabiata*, and the nuclear genes (Myh6 and sRH1) of the two species were not fully differentiated.

The phylogenetic patterns of mitochondrial and nuclear DNA markers of plateau loach fish in the Qilian Mountains are generally consistent. Highly differentiated lineages and expansion signals indicate that multiple small, isolated populations are persistent in adverse environmental conditions. This finding supports the predictions of “A refuge in a refuge area” models established in other plant and animal groups (Dufresnes et al., 2016; Gómez & Lunt, 2007). As the intersection area of the ancient Yellow and Tarim River systems, the Qilian Mountain area was influenced by the geological uplift of the Qinghai‐Tibet Plateau many times and formed the existing water system. In the course of strong geological structure and river development, *T. hsutschouensis* colonized from east to west, while *T. papillosolabiata* colonized from west to east along the northern slope of the Qilian Mountains. The effects of ice age and geographical isolation promoted the genetic differentiation of plateau loach and even formed some endemic species (Feng et al., 2017; Lv et al., 2020; Wu et al., 2013). Thus, these factors laid the foundation for the current pattern of species distribution.

### 4.2 Cryptic diversity

Combined with the degree of delicacy in species differentiation of plateau loach *Triplophysa* (He et al., 2011; Wang et al., 2020), the existence of a highly differentiated lineage of plateau loach in the Qilian Mountains is consistent with the existing morphological taxonomy (Zhu, 1989). In the phylogenetic tree of mtDNA, 11 species have formed their own phylogenetic clades, and *T. leptosoma* and *T. papillosolabiata* are sister groups with great morphological differences (Wang, 1991; Zhao & Wang, 1983). *Triplophysa leptosome* mainly lives in environments with fine‐flowing water, while *T. papillosolabiata* generally lives in environments with deep water and slow‐flowing water. From the perspective of the living environment, they have their own unique ecological niches. As seen from the structure diagram based on microsatellite data, *T. leptosoma* was differentiated from the Changma or Danghe populations of *T. papillosolabiata*, which also corresponds to the geographical location of the specimen collection. The MJN map of the nuclear genes showed that *T. leptosoma* and *T. papillosolabiata* shared the same haplotype in sRH1 and Myh6, while the two species had their own unique haplotype in the nuclear gene RAG1, which indicated that the differentiation time of *T. leptosoma* and *T. papillosolabiata* was relatively young.

As seen from the structure diagram, when the K value was 11, *T. papillosolabiata* could be divided into different groups according to water system (except that the Suganhu population merged into the population of the Hei River system), but *T. hsutschouensis* does not show a similar phenomenon, which may be related to the strong exotic evolution ability of the plateau loach (Zhao, 1984). When the *K* value was 14, the Heihe population of *T. papillosolabiata* could be divided into four different groups: Babahe, Linze, Yanglong, and Suganhu populations. At the same time, there was an obvious gene introgression between the Shiyanghe populations of *T. papillosolabiata* and *T. wuweiensis*. Interspecies hybridization and gene exchange exist widely in nature, and this hybridization process plays an important role in the evolution of many species (Abbott et al., 2013; Arnold & Meyer, 2006). In the phylogenetic tree of mtDNA, the Shiyanghe and Heihe populations formed a large branch and formed a sister group with the Shulehe population. According to the genetic differentiation index, the genetic differentiation between the Suganhu and Shiyanghe populations was the largest, while the genetic difference between adjacent populations was relatively small, which was related to the geographical distance between the two populations. The AMOVA of different populations of *T. papillosolabiata* showed that 82.06% of the molecular differences were within the populations. In the MJN map of nuclear genes, the phenomenon of different populations divided according to drainage patterns was also not supported by the three nuclear loci. These results indicate that a genetic differentiation trend is emerging in the population of *T. papillosolabiata*.

### 4.3 Species differentiation and drainage history

The genetic differentiation and species origin of plateau fish are closely related to the uplift and climate change of the Tibetan Plateau (He et al., 2006). It is important to consider the differentiation time of the *Triplophysa* in the Tibetan Plateau in light of geological events. According to strict molecular clock estimation, He et al. (2006) concluded that the time of plateau loach differentiation from other loaches was approximately 13.5–10.3 MYA. Wang et al. (2012) combined Cyt*b* and D‐loop sequence analysis and proposed that *Triplophysa rosa* differentiated from the genus *Triplophysa* at approximately 48.3 MYA. Wang et al. (2016) found that the differentiation time of plateau loach from the Cobitidae family by the mitochondrial genome was approximately 23.5 MYA. Our estimate of the differentiation time of plateau loach (19.4 MYA) is closer to the molecular clock estimate of Wang et al. (2016).

The Shiyang River system belongs to the Wuwei Basin, which was connected with the ancient Yellow River system long ago. In the Miocene, the uplift of the Wushaoling Mountains blocked the connection between the Shiyang River system and the ancient Yellow River (Feng, 1963). Some *T. stoliczkae* and *T. robusta* formed remnant populations in the Gulang River, a tributary of the Shiyang River, and survive to this day due to the uplift of mountains. In the process of *T. papillosolabiata* colonization from west to east, the genetic differentiation of the Heihe population was large under the influence of geological events in the Qilian Mountains (Chen et al., 2010; Zhou et al., 2013). There was no differentiation between the Yanglong population in the upper reaches of the Taolai River, the largest tributary of the Hei River, and the Linze population in the middle reaches of the Hei River. Due to the influence of climate, the water temperature in the lower reaches of the Taolai River is relatively high, and no plateau loach samples have been collected. However, these two populations diverged from the Babao population in the upper reaches of the Hei River, and the Suganhu population also diverged from the Heihe population. Combined with the historical dynamics of *T. papillosolabiata* population differentiation, we speculated that at the beginning of the formation of the Hei River system, the ancient Hei River was composed of the Taolai River as the upper reaches and the middle and lower reaches of the Hei River were formed by other seasonal rivers. The Yeniugou section of the upper reaches of the Hei River is connected with the Datong River. Influenced by the uplift of the Tolle Mountains, the Yeniugou River cut down the Corridor Nanshan into the Hexi Corridor and eventually merged into the ancient Hei River, which was consistent with the geological analysis results (Feng, 1963). The Suganhu population may have been formed by the Yanglong population in the upper reaches of the Taolai River, which was connected with the Harteng River due to river capture in the interglacial period. The Harteng River is the main water source of Sugan Lake. It is generally believed that there are three relatively independent river systems in the Hexi Corridor, namely, the Shiyang, Hei, and Shule River systems, and the Sugan Lake and Harteng River systems are merged into the Shule River system. According to the population differentiation of *T. papillosolabiata*, the Sugan Lake system was never historically connected with the Shule River system but was connected with the Taolai River in the upper reaches of the Heihe River.

### 4.4 Implications for conservation

Population genetic diversity is an important part of biodiversity. The level, formation mechanism, and distribution pattern of genetic diversity can reveal the origin and evolution of species and are also the basis of species conservation. The maintenance of genetic diversity prevents the loss of the evolutionary potential of species (Bartáková et al., 2019; ). The genetic differentiation characteristics of the plateau loach fish in the Qilian Mountains play an important role not only in fish conservation but also in water ecological stability in the Qilian Mountains of China. According to the principle of setting evolutionarily significant units (ESUs) and management units (MUs) (Frankham, 2003; Moritz, 1994), our analysis shows that due to the unique genetic characteristics of *T. shiyangensis* and *T. hsutschouensis*, including exclusive mtDNA haplotypes and nuclear alleles, they need to be protected as conservation and management units. *T. wuweiensis* and *T. leptosoma* are highly differentiated and unique populations with low genetic diversity and narrow distribution areas and require priority protection.

The unique ecological environment on the northern slope of the Qilian Mountains has bred some unique species of plateau loach, but its ecological environment problems deserve special attention. Global warming has led to the extinction of loach fish in the lower reaches of the Taolai River, and several seasonal rivers (except the Liyuan River) have long dried up. The distribution areas of *T. shiyangensis* and *T. wuweiensis* are mainly in the tributaries of the upper reaches of the Shiyang River, and the construction of artificial irrigated areas has severely squeezed the habitat space of fish (Ma et al., 2005). The protection of plateau loach fish in the Qilian Mountains should focus on the protection of habitat; the protection of vegetation on both sides of the river is also very important, as it can stabilize the riverbed and reduce flood disturbance. The genetic differentiation of loach species in this study can provide guidance for subsequent conservation work.

## 5 CONCLUSIONS

In this study, we evaluated the genetic differentiation of the genus *Triplophysa* in the Qilian Mountains by integrating mitochondrial gene, nuclear gene, and microsatellite data. There is a high degree of lineage differentiation among the species, and *T. leptosoma* is a new species undergoing differentiation. The tectonic events of the uplift of the Qinghai‐Tibet Plateau and the repeated climatic fluctuations during the Quaternary glaciation had a great influence on the genetic structure of the plateau loach in the Qilian Mountains, which promoted the genetic differentiation of the plateau loach and led to the formation of some endemic species. *T. hsutschouensis* colonized from east to west, and population expansion occurred during the last glacial period of the Qinghai‐Tibet Plateau, while *T. papillosolabiata* colonized from west to east along the northern slope of the Qilian Mountains. There were two kinds of colonization paths in opposite directions in the Qilian Mountains, which formed the present species distribution pattern. In addition, habitat conservation is necessary for species with low genetic diversity and narrow distribution (*T. wuweiensis* and *T. leptosoma*) to ensure the persistence of genetic diversity. Combined with genetic evidence from other species in the region, our results suggest that different biogeographic patterns emphasize the importance of the Qilian Mountains as a research hotspot for the genetic diversity of some plant and animal groups.

## AUTHOR CONTRIBUTIONS

**Yanyan Du:** Formal analysis (equal); investigation (equal); resources (equal); software (equal); writing – original draft (lead). **Yanping Zhang:** Investigation (equal). **Zhongyu Lou:** Formal analysis (equal); software (equal). **Tai Wang:** Data curation (lead); investigation (equal); methodology (equal); writing – original draft (equal); writing – review and editing (equal).

## FUNDING INFORMATION

This work was supported by the National Natural Science Foundation of China (Project 31460560); Innovation Base and Talent Program of Gansu Province (No.21JR7RA720). The funding bodies played no role in the design of the study and collection, analysis, and interpretation of data and in the writing of the manuscript.

## CONFLICT OF INTEREST STATEMENT

The authors declare that they have no competing interests.

## Data Availability

The raw data underlying the main results of the study are archived in GenBank (accession numbers: OQ359293–OQ359372, OQ360026–OQ360052, OQ363686–OQ363749, OQ376574–OQ376597).
